# Resurrection of Ancestral Malate Dehydrogenases Reveals the Evolutionary History of Halobacterial Proteins: Deciphering Gene Trajectories and Changes in Biochemical Properties

**DOI:** 10.1093/molbev/msab146

**Published:** 2021-05-11

**Authors:** Samuel Blanquart, Mathieu Groussin, Aline Le Roy, Gergely J Szöllosi, Eric Girard, Bruno Franzetti, Manolo Gouy, Dominique Madern

**Affiliations:** 1 Univ Rennes, Inria, CNRS, IRISA, Rennes, France; 2 Université Lyon 1, CNRS, UMR5558, Laboratoire de Biométrie et Biologie Évolutive, Villeurbanne, France; 3 Center for Microbiome Informatics and Therapeutics, Massachusetts Institute of Technology, Cambridge, MA, USA; 4 Univ Grenoble Alpes, CNRS, CEA, IBS, Grenoble, France; 5 MTA-ELTE “Lendulet” Evolutionary Genomics Research Group, Budapest, Hungary

**Keywords:** ancestral protein resurrection, horizontal gene transfer, halophilic adaptation of proteins, malate dehydrogenase, Haloarchaea, Archaea

## Abstract

Extreme halophilic Archaea thrive in high salt, where, through proteomic adaptation, they cope with the strong osmolarity and extreme ionic conditions of their environment. In spite of wide fundamental interest, however, studies providing insights into this adaptation are scarce, because of practical difficulties inherent to the purification and characterization of halophilic enzymes. In this work, we describe the evolutionary history of malate dehydrogenases (MalDH) within Halobacteria (a class of the Euryarchaeota phylum). We resurrected nine ancestors along the inferred halobacterial MalDH phylogeny, including the Last Common Ancestral MalDH of Halobacteria (LCAHa) and compared their biochemical properties with those of five modern halobacterial MalDHs. We monitored the stability of these various MalDHs, their oligomeric states and enzymatic properties, as a function of concentration for different salts in the solvent. We found that a variety of evolutionary processes, such as amino acid replacement, gene duplication, loss of MalDH gene and replacement owing to horizontal transfer resulted in significant differences in solubility, stability and catalytic properties between these enzymes in the three Halobacteriales, Haloferacales, and Natrialbales orders since the LCAHa MalDH. We also showed how a stability trade-off might favor the emergence of new properties during adaptation to diverse environmental conditions. Altogether, our results suggest a new view of halophilic protein adaptation in Archaea.

## Introduction

The adaptation of organisms to fluctuating environments occurred through two evolutionary processes: amino acid replacements (replacement process, RP) that affect protein structure, function and dynamics (Tomatis et al. 2008; Liu and Bahar 2012), and, less frequently, horizontal gene transfer (HGT, the acquisition of a foreign functional coding gene), which can quickly modify the properties of an organism (Fournier et al. 2015). With respect to RP, a modern protein has evolved on a trajectory, through a series of now extinct ancestral states, via gradual primary sequence modifications. Sequence variability is shaped by the equilibrium between genetic drift and Darwinian selection, which will tend to clean out most of the mutations in order to maintain initial structure and function (Tokuriki and Tawfik 2009a). HGT has been detected in numerous organisms and is well documented as an important driving evolutionary force in prokaryotes and eukaryotes (Papke et al. 2004; Khomyakova et al. 2011; Andam et al. 2012; Naor et al. 2012; Kaminskiet al. 2013; Alexander et al. 2016). However, experimental studies that determine the properties of HGT genes are very rare (Savory et al. 2018). Here, we report the recovery and analysis of protein evolutionary trajectories, impacted both by gradual RP and HGT, which preserved structure and function while adapting to extreme physicochemical conditions.

A protein is a dynamical entity, which exists as a population of conformers (alternative substructures having slightly different free energies) represented statistically in specific proportions on various time scales (Frauenfelderet al. 2009; Lewandowski et al. 2015). Protein conformational landscapes are determined by hydrophobic, ionic, hydrogen bond and Van der Waals interactions between residues within the macromolecule as well as between the macromolecule and its solvent environment. Solvent interactions, in particular, influence folding pathways, stability, dynamics and solubility of the native state, so that proteins can be considered as dynamic protein–solvent complexes (Svergun et al. 1998; Bellissent-Funel et al. 2016). Mutational processes, therefore, impact protein–solvent interactions as well as intramolecular interactions. So far, however, we have not been aware of published work on the effects of amino acid replacements on protein–solvent interactions along an evolutionary trajectory. In order to address this fundamental question, we adopted halobacterial MalDH as a model enzyme and applied ancestral protein sequence resurrection (ASR) to access ancient proteins on the evolutionary pathway, in order to compare their properties with those from extant MalDH orthologs. ASR has been intensively used to investigate various functional changes in proteins and phenotypic adaptations to environmental constraints (Gaucher et al. 2003; Finnigan et al. 2012; Hobbs et al. 2012; Voordeckers et al. 2012; Mirceta et al. 2013; Hart et al. 2014; Kacar et al. 2017; Gonzales-Ordenes et al. 2018). MalDH is a ubiquitous enzyme involved in central metabolism, of which several forms have been characterized. In Archaea, MalDH is a constitutively expressed enzyme, which operates in the tri-carboxylic acid (Krebs) cycle to reversibly catalyze the conversion of malate into oxaloacetate linked to the oxidation/reduction of dinucleotide coenzymes (Madern 2002; Minárik et al. 2002).In order to maintain their efficiency within the metabolism, MalDHs are adapted to compete against the harsh conditions frequently encountered in extremophilic Archaea.

Here, we chose to investigate adaptation to high salinity. Recent phylogenetic studies have shown that the “salt-in” strategy used for thriving in hypersaline environments may have independently emerged on three occasions in Archaea (Aouad et al. 2018, 2019). Amongst these organisms, Halobacteria (a class belonging to Euryarchaeota), also frequently mentioned as Haloarchaea, are the most extensively studied (Oren 2002). Species from Halobacteria are adapted primarily to molar salt environments, with some species displaying secondary adaptation to other extreme physicochemical conditions, such as high pH (Falb et al. 2005), high (Kim et al. 2018), low temperature (DasSarma et al. 2013), or high pressure (Albuquerque et al. 2012). Early work suggested that halobacterial species obey the salt-in strategy, that is, the accumulation of high concentrations of mainly KCl in the cytoplasm to counterbalance the high osmotic pressure due to NaCl outside (Oren 2002). Analyses of ion transporter gene distribution have revealed, however, that the intracellular composition is better described as a complex, dynamic mixture of various salts and compatible solutes in order to accommodate salinity fluctuations encountered in the natural environment (Becker et al. 2014). The specific intracellular composition imposes further adaptation at the protein level to maintain solubility, stability and activity for a range of saline conditions (Madern, Ebel, Zaccai, et al. 2000).

Numerous studies have used tetrameric NADH-dependent MalDH from halophilic Archaea and Bacteria as a model to describe how solvent interactions govern protein properties (Costenaro et al. 2002; Irimia et al. 2003; Coquelle et al. 2010; Talon et al. 2014). Because HGT has been reported as a frequent phenomenon in Halobacteria (Papke et al. 2004; Khomyakova et al. 2011) and because some halobacterial MalDHs result clearly from duplication events, we performed ASR taking into account HGT events. In order to ensure maximum accuracy, we considered advanced phylogenetic models and large taxonomic samples; we estimated the halobacterial species phylogeny by analyzing simultaneously core genes, and inferred a joint MalDH phylogeny accounting for HGTs, duplications and losses. In addition, we applied indel-aware models to estimate ancestral MalDH sequences. Finally, we synthesized a number of key ancestral MalDHs.

Salt-dependent conformational stability and enzymatic properties of MalDHs from different modern lineages, together with their resurrected ancestors, were determined, by applying the same methodology as in our previous studies of the MalDH from *Haloarcula marismortui.* Furthermore, analytical ultracentrifugation (AUC) experiments demonstrated how changes of the oligomeric state equilibrium between active conformers could rescue the deleterious effect of low salt concentration in some lineages.

These are the main findings of our study: The last common ancestor MalDH of Halobacteria (LCAHa) does not necessitate multi-molar KCl in order to be stable and active, suggesting it was mildly halophilic; the paleo environment at the origin of Halobacteria in Archaea could not be inferred, because the KCl-concentration-dependent stabilities of MalDHs are not correlated with the respective environmental conditions of their hosts; the description of a tradeoff mechanism by which the haloalkaliphilic lineage of MalDH has emerged; and the unexpected experimental outcome that the modern MalDH from *H. marismortui* (the most studied halophilic enzyme to date) was the result of an ancestral HGT from an early haloalkaliphilic ancestor.

We concluded that solubility, stability and activity of MalDHs facing extreme salt conditions did not evolve concomitantly, and discuss, here, how these findings establish a new understanding of halophilic adaptation in MalDHs.

## Results

### Phylogenetic Inferences

**Fig. 1.**
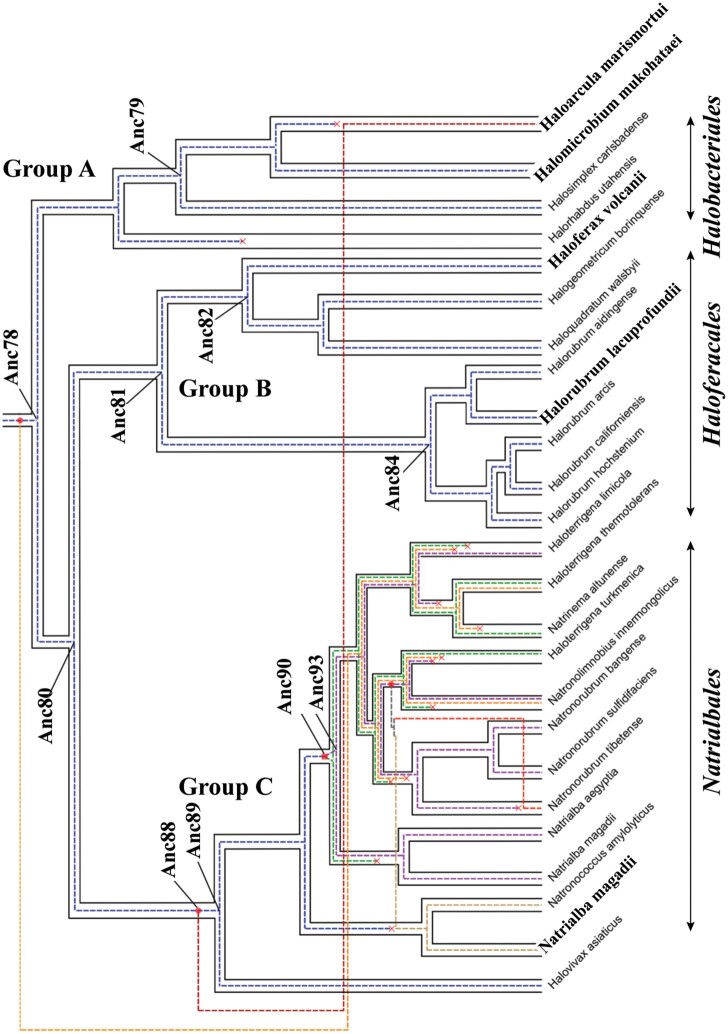
Halobacteria species tree (pipes) and MalDH gene tree (dashed colored lines). The species tree is subdivided into three sublineages, groups A–C. Biochemical characterization of extant MalDHs were obtained for *Haloarcula marismortui* and *Halomicrobuim mukohataei* (group A), *Haloferax volcanii* and *Halorubrum lacusprofundi* (group B), and *Natrialba magadii* (group C). The halobacterial MalDH genes have encountered a complex history, with gene loss (crosses), gene transfers (dots), and gene duplication (squares). The ten ancestral MalDH sequences for which biochemical properties were studied are indicated on the MalDH tree nodes (Anc “N”). The examined ancestral MalDH existed in the last common ancestor of Halobacteria (LCAHa, Anc78), in LCAs of group A (Anc79), group B plus C (Anc80), group B (Anc81), and group C (Anc89). We also examined recent ancestors of *H. volcanii* (Anc82) and *H. lacusprofundi* (Anc84) MalDHs within group B, the MalDH ancestors of paralogous duplicates (Anc90) and of the copy inherited in *N. magadii* (Anc93) within group C, and finally the LCA (Anc88) of the transferred *H. marismortui* MalDH and of group C MalDHs.

Groussin et al. (2015) have shown the importance of taking into account the process of diversification within prokaryotes, in which both RP and HGT are accounted for when performing ASR. First, the species tree of Halobacteria was reconstructed from a large phylogenomic concatenation (see Materials and Methods). We examined several sources of reconstruction artifacts resulting from long branch attraction (LBA) artifacts and compositional biases to obtain a robust and strongly supported halobacteria species phylogeny (fig. 1 and supplementary fig. S1, Supplementary Material online). Halobacteria appeared to be subdivided into three main sub-lineages: Halobacteriales (group A, including *H. marismortui:* “*H. mari*” and *Halomicrobuim mukohataei:* “*H. muko*”), Haloferacales (group B, including *Haloferax volcanii:* “*H. volc*” and *Halorubrum lacusprofundi:* “*H. lacu*”) and Natrialbales(group C, a monophyletic group of alkaliphilic halobacterial species adapted to high pH environments and including *Natrialba magadii:* “*N maga*”), consistent with previous findings (Brochier-Armanet et al. 2011). Moreover, we inferred a strongly supported monophyly of species from groups B and C (see Supplementary Material section “Species tree reconstruction”).

In the second step of the analysis, a MalDH tree was reconstructed using the site heterogeneous Bayesian phylogenetic model CAT+GTR (Lartillot et al. 2009) (see Materials and Methods and supplementary material and fig. S2, Supplementary Material online). It reveals a weak phylogenetic signal and large incongruities with respect to the Halobacteria species phylogeny (supplementary fig. S1, Supplementary Material online).

Then, the reconstructed MalDH tree was reconciled with the species tree of Halobacteria (see Materials and Methods), yielding a final MalDH phylogeny correcting initial stochastic uncertainties and accounting for gene duplication, horizontal transfers and losses (DTL, Szöllosi, Rosikiewicz, et al. 2013; Szöllosi, Tannier, et al. 2013). This phylogeny, called the MalDH “joint-tree,” is presented in supplementary figure S3, Supplementary Material online and figure 1. Importantly, the MalDH joint-tree appears to be well supported (see posterior probabilities [PP] in supplementary fig. S3, Supplementary Material online). It reveals a highly supported HGT toward *H. marismortui* from an ancestral lineage related to the ancestor of group C species (PP = 0.99). This strong support, the short length of the *H. mari* MalDH branch (0.086 expected replacement per site, see supplementary fig. S3, Supplementary Material online) and biochemical observations (see below, enzymatic characterization) suggest that this clustering unlikely results from a LBA. The tree also describes a complex history of MalDH DTL events within the alkaliphilic Halobacteria (group C, see supplementary fig. S3, Supplementary Material online). No other DTL events are inferred during the evolution of MalDHs in groups A and B (fig. 1).

Subsequently, the MalDH protein sequences were aligned anew using PRANK (Löytynoja and Goldman 2008), considering indels as evolutionary events and the MalDH joint-tree as guide tree (see Materials and Methods).

Finally, the ancestral sequences were computed from the PRANK alignment, considering the MalDH joint-tree as a fixed topology, and using the CAT+GTR Bayesian model (Lartillot et al. 2009, see Materials and Methods). Control experiments were performed using other available methods, implemented in the Maximum Likelihood framework, in order to assess the accuracy of the ancestral sequence reconstructions (ASRs). Most noticeably, in each ten examined ancestral MalDHs, at least 90% of ancestral amino acid sites appear well identified (PP > 0.9), and only 2–6 sites are differently inferred depending on the applied method (see Supplementary Material, section “Ancestral MalDH reconstruction”). The MalDHs retained for biochemical characterizations span the oldest ancestors in Halobacteria and several recent ancestors close to the five extant MalDHs with known properties (fig. 1).

### Evolution of Surface Residues and Protein Solubility

Acidic residue enrichment of proteomic composition in halophilic species, when compared with nonhalophiles is well established (Paul et al. 2008).The electrostatic field generated by charges and the solvent-accessible hydrophobic surface of a halophilic protein can be calculated using its crystal structure (Coquelle et al. 2010). These calculations were applied to a set of 15 structures of halophilic proteins, compared with their nonhalophilic counterparts. They showed that the negatively charged amino acid enrichment is a specific feature of the solvent-accessible surface that is compensated by a decrease in basic residues (Graziano and Merlino 2014).

**Fig. 2.**
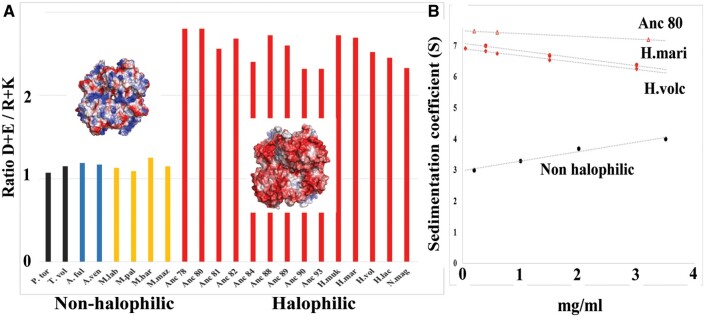
Acidic amino acid enrichment favors solubility in high KCl concentration. (*A*) The ratio between negatively charged residues over positively charged ones (DE/KR) for various modern and ancestral MalDH. High values of the ratio reflect acidic amino acid surface enrichment as it is illustrated by the electrostatic surface representation (small insets) of *Haloarcula marismortui* MalDH (pdb code 4J5K) and *Picrophilus torridus* MalDH (pdb code 4BVG). The positively and negatively charged surfaces are colored in blue and red, respectively. (*B*) Variations of the experimental sedimentation coefficient (s exp) at 3.8 M KCl, recorded at various protein concentrations, for Anc80 MalDH and *H. volc* MalDH (this work). Data for *H. mari* MalDH and for a nonhalophilic MalDH are from Coquelle et al. (2010).

Studies comparing MalDHs from extremely halophilic Bacteria and Archaea with nonhalophilic homologs have clearly demonstrated that this change in surface electrostatic properties is an efficient strategy to maintain protein solubility at high salt concentration (Solovyova et al. 2001; Coquelle et al. 2010; Talon et al. 2014). We calculated the ratio between negatively (D + E) and positively (R + K) charged amino acids for several modern and ancestral MalDHs. DE/KR ratios >2 indicate that the acidic amino acid surface enrichment is a common property of halobacterial MalDH shared since the root node Anc78 (five modern species, nine reconstructed MalDH ancestors characterized), whereas the nonhalophilic outgroup of modern MalDH displays nearly neutral surfaces (fig. 2*A*). A recent study of salt adaptation in Methanosarcinales has shown that the surface of ADP-dependent kinases changes its amino acid composition already in the moderately high environmental concentration of 0.5 M NaCl (Gonzales-Ordenes et al. 2018). The DE/KR ratio we calculated using Methanosarcinales MalDHs also indicates a moderate acidic enrichment (ratio of 1.5, supplementary table S4, Supplementary Material online). This suggests that, among other mechanisms, protein surface acidic enrichment could be a widespread strategy in moderate to extreme halophilic adaptation. According to most recent molecular phylogenies however, Halobacteria are closer to Methanomicrobiales than to Methanosarcinales (Aouad et al. 2018). Consequently, understanding if these phenotypes are convergent or diverged gradually from a common origin would deserve further scrutiny, which falls outside the scope of this study.

In previous studies on *H. mari* MalDH, we have found that under given solvent conditions, constant or slightly decreasing sedimentation coefficient values over increasing protein concentration indicated interparticle repulsive effects favoring solubility of the tetrameric enzyme. In contrast, an increasing relation would indicate the general tendency of the protein to aggregate at high salt concentration (Solovyova et al. 2001; Coquelle et al. 2010). We applied the same methodology on the MalDH of *H. volcanii* and Anc80 in 3.8 M KCl. The negative slopes observed in figure 2*B* for ancestral and modern halobacterial MalDHs confirm that they are soluble as tetramers at high KCl concentration, whereas, under similar conditions, nonhalophilic proteins precipitate. To conclude, our data showed that acidic surface enrichment is an ancestral mechanism in Halobacteria to avoid protein precipitation in high salt conditions.

### Salt Concentration Dependence of Conformational Stability in Modern and Ancestral MalDHs

Depending on their effects on the conformational stability of proteins, salts have been categorized into three mains groups: “salting-out,” “neutral,” and “salting-in” (Von Hippel and Schleich 1969). In a pioneering work, the effects of salts from these groups on of *H. mari* MalDH suggested that stability mechanisms accommodate to solvent conditions (Bonneté et al. 1994). The study revealed the additivity of stabilization forces in this halophilic protein, their subtle equilibria, as well as the rich palette of energy barriers competing against deleterious physico-chemical conditions. Since, numerous biophysical studies on the effects of physiological and nonphysiologically relevant salts, aimed at understanding halophily in *H. mari* MalDH, have revealed that stability strongly depends on the nature of solvent ions (Bonneté et al. 1994, Madern and Zaccai 1997; Ebel et al. 1999; Irimia et al. 2003; Madern and Ebel 2007).In particular, they have shown that ion charge density is the major determinant influencing the unfolding transition when the salt concentration decreases: higher ion charge densities induce stronger electrostatic interactions resulting in a stable protein fold in low salt concentration. Cation stabilizing efficiency of the folded form follows the series Ca^2+^ and Mg^2+^ stronger than K^+^, Na^+^, Li^+^, and ${\text{NH}}_{4}^{+}$ stronger than Rb^+^, Cs^+^. The corresponding anion series is ${\text{SO}}_{4}^{2–}$, OAc^–^, and F^–^ stronger than Cl^–^ stronger than Br^–^ and I^–^.

Here, we applied the same comparative strategy using different salts to investigate in detail how stability evolved in various ancestral and modern MalDHs. The strategy offered the opportunity to understand fundamental processes enabling protein stability in various salts and to reveal the unsuspected effect of the various ions that, additionally to K^+^ and Cl^–^, populate the cytoplasm of halophilic cells. Hence, in this article, *stability* and *stabilizing effects* will refer to the protein propensity to remain folded in low salt concentrations, whereas *instability* and *destabilizing effects* will preclude protein folding in such conditions.

Supplementary figure S5, Supplementary Material online shows the salt concentration dependent stability of modern MalDHs, from *H. marismortui*, (*H. mari*) and four newly characterized species, *Haloarcula mukohatei* (*H. muko*)*, N. magadii* (*N. maga*), *H. volcanii* (*H. volc*), and *H. lacusprofundii* (*H. lacu*), as well as from nine of their ancestors. In KCl, all the ancestral and modern enzymes display folded-unfolded transitions that are shifted toward lower concentration compared with *H. mari* MalDH (supplementary fig. S5*B*, Supplementary Material online). These stability curves help to determine the *M^f^*_½_ parameter, which indicates the salt concentration at which 50% of a given halophilic MalDH is still folded and active.

Consequently, we show that multi-molar salt concentration is not mandatory for the stabilization of a halobacterial enzyme, in contrast to what has long been assumed. Our data, which present the first characterization of the salt requirements of an enzyme from various extant halophilic species, also clearly established that there is no correlation between *M^f^*_½_ values in KCl and NaCl salt concentration in the environment (table 1).The shift of folded-unfolded transition curves toward lower salt concentration in KF (supplementary fig. S5*A*, Supplementary Material online) compared with KCl (supplementary fig. S5*B*, Supplementary Material online) indicates that the stabilizing effect induced by a high charge density anion acts on all ancestral and modern enzymes. Reciprocally, when Cs^+^, a weaker stabilizing cation, is used, all the modern and ancestral MalDH transition curves are shifted toward higher concentration, showing destabilization effects (supplementary fig. S5*C*, Supplementary Material online).

**Table 1.** NaCl Concentration of the Considered Species Environment (Oren 2002) and *M^f^*_½_ in KCl of the Corresponding MalDHs.

Current Species	[NaCl] of the Medium	KCl *M^f^*_½_ of MalDH
*Haloferax volcanii*	2 M	0.30 M
*Haloarcula mukohatei*	4 M	0.35 M
*Halorubrum lacusprofundii*	4 M	0.30 M
*Natrialba magadii*	4 M	0.80 M
*Haloarcula marismortui*	4 M	1.40 M

Our results, moreover, show that the oldest considered ancestor of halobacterial MalDH (Anc78 MalDH) was a halophilic enzyme requiring salt to remain properly folded. This enzyme is stable between 0.8 M and 3.8 M KCl, with an *M^f^*_½_ value of 0.5 M (supplementary fig. S5*B*, Supplementary Material online). Compared with the *M^f^*_½_ value of 1.4 M for *H. mari* MalDH, considered as an obligate halophilic enzyme, Anc78 MalDH may be considered as moderately halophilic.

### Distinct Evolutionary Trajectories on the Way to Modern Halobacterial MalDHs

**Fig. 3.**
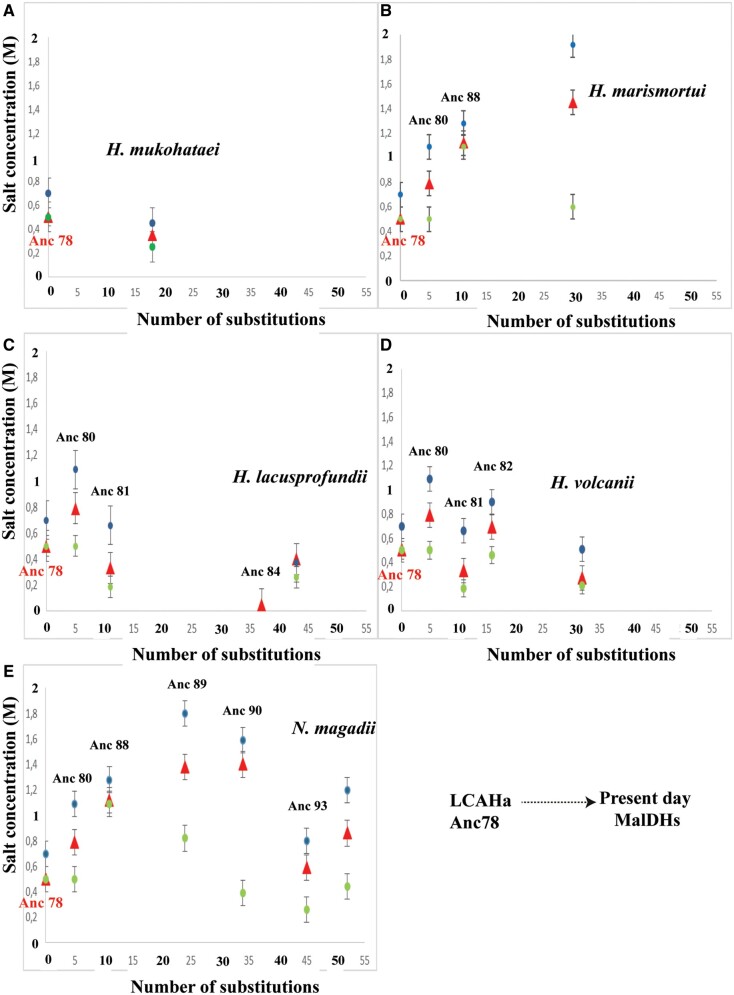
Evolution of the conformational stability of ancestral and extant halobacterial MalDHs. The *M^f^*_½_ values (*y*-axis) are plotted against the number of amino acid replacements accumulated since the LCAHa Anc78 MalDH (*x*-axis). Values were recorded in KCl (red), CsCl (blue), and KF (green). (*A* and *B*) Evolutionary path leading to two extant MalDH enzymes of group A species, *H. muko* and *H. mari* MalDHs, respectively. (*C* and *D*) Evolutionary path leading to two extant MalDH enzymes of group B species, *H. lacu* and *H. volc*. (*E*) Evolutionary path leading an extant MalDH enzyme of a group C species *N. maga*.

Contrary to *H. mari* MalDH requiring high salt concentrations in order to be stable, five modern MalDH and their ancestors display various stability phenotypes in low salt. In this work, resurrected ancestral MalDHs provided insights into the evolution of these phenotypes. In previous work on paleo-environmental temperatures, $T_{½}^{M}$ (the temperature at which 50% of the resurrected protein is still folded) have been plotted as a function of ancestor age (Gaucher et al. 2003). We applied, here, an analogous approach, by plotting *M^f^*_½_ values (determined using supplementary fig. S5, Supplementary Material online) versus the number of amino acid replacements accumulated from the oldest considered Anc78 MalDH. The number and nature of these replacements were determined from a sequence comparison between ancestral and modern enzymes (supplementary figs. S6–S8, Supplementary Material online). The results for physiological KCl (fig. 3, red triangles) show that MalDHs evolutionary trajectories differ over the distinct considered lineages.

In group B, MalDHs encountered no DTL event and all reconstructed ancestral MalDHs are orthologs, transmitted vertically during species evolution. The *M^f^*_½_ values of modern MalDH (*H. lacu* and *H. volc*) in this group are similar to the *M^f^*_½_ value of theroot LCAHa Anc78 MalDH (fig. 3*C* and *D*). Varying stability of intermediate ancestral MalDHs (Anc80, Anc81, Anc82, and Anc84, see fig. 3) suggest genetic drift, where some destabilizing replacements are counterbalanced by other stabilizing replacements, leading to nearly neutral evolution with respect to protein stability. This genetic drift hypothesis might be further tested using Dn/Ds ratio measurement, providing that higher taxonomic sampling and DNA sequences could be analyzed. This however stands out of the scope of this study focusing on protein and ASR phenotypes characterization. Within group B, the unexpected case of Anc84 MalDH, a close ancestor of *H. lacu* MalDH, is of great interest: the protein shows non-obligate halophilic behavior and is stable at very low KCl concentration (0.064 M). This suggests a peculiar evolution pattern (see below).

In group C, the first part of the evolutionary trajectory toward *N. maga* MalDH is strongly destabilizing (fig. 3*E*), with KCl *M^f^*_½_values increasing drastically between Anc78 MalDH and Anc90 MalDH, the ancestor of two paralogous MalDH duplicates in group C (fig. 1). However, in the second part of the trajectory, *M^f^*_½_ indicate greater protein stability in relatively low KCl concentration for Anc93 MalDH (one of the paralogous copies deriving from Anc90) and *N. maga* MalDH (deriving from Anc93), with the latter modern MalDH being slightly less stable than Anc78 MalDH.

In group A, *H. muko* MalDH has followed the shortest evolutionary trajectory since the root Anc78 MalDH, diverging by 18 amino acid replacements (fig. 3*A*). Its KCl *M^f^*_½_ values are similar to the ones recorded for Anc78 MalDH, and very distinct from the ones recorded for the *H. mari* MalDH, despite the fact that *H. muko* and *H. mari* are two related species in group A (fig. 1). It has been assumed that *H. mari* MalDH reflected typical behavior for a protein facing extreme environmental salt concentration (Madern, Ebel, Zaccai, et al. 2000). Our results now challenge this point of view, by showing that the low conformational stability of *H. mari* MalDH is not a phenotype shared by other modern MalDHs. Moreover, our phylogenetic inferences suggest that *H. mari* MalDH is highly divergent when compared with the LCAHa Anc78 MalDH, and that it most likely evolved by HGT from an extinct lineage related to the ancestor of the alkaliphilic group C (fig. 1). *H. mari* MalDH has diverged by 22 amino acid replacements from Anc88, its common ancestor with group C MalDHs, while retaining some of its properties, such as high instability (see also below).

Importantly, MalDH stability phenotypes were shown to depend, not only on the salt concentration, but also on the salt type. For example, in previous studies on *H. mari* MalDH, it has been found that the replacement of K^+^ by another monovalent cation is always destabilizing. We observed that this conclusion holds also for ancestral and modern MalDH. This suggests that the salt concentration dependence of halobacterial MalDH stability relies on a common ion-linked mechanism, shared since the root ancestor, Anc78.

### Heterogeneous Stability Phenotypes of Modern and Ancestral MalDHs Are Modulated by Ions of High Charge Density

According to our previous studies, ions of high charge density enhance the stability of *H. mari* MalDH at low salt concentration (Madern and Zaccai 1997; Ebel et al. 1999; Irimia et al. 2003; Madern and Ebel 2007). We investigated how this observation applies to the various studied MalDHs, in different salts involving ions of higher charge density than Cl^–^ and K^+^.

#### Anion Effects

We explored the effect of high charge density anions by comparing stability in KF and KCl. In contrast to CsCl, KF *M^f^*_½_ trajectories for the different proteins do not vary consistently when compared with KCl. *M^f^*_½_ values for the two salts are similar for Anc78, Anc88, Anc84 MalDHs, and H. *volc* MalDH. KF is slightly more stabilizing for Anc81 MalDH, *H. muko*, and *H. lacu* MalDHs. Most interestingly, KF is strongly stabilizing when compared with KCl not only for Anc80 MalDH and *H. mari* MalDH (fig. 3*B*), but also within group C, for Anc89, Anc90, Anc93 MalDHs, and *N. maga* MalDH (fig. 3*C*). This suggests particular phenotypes for some modern and ancestral MalDH related to group B and C species, where a higher charge density anion tends to favor stability in low salt concentration.

**Fig. 4.**
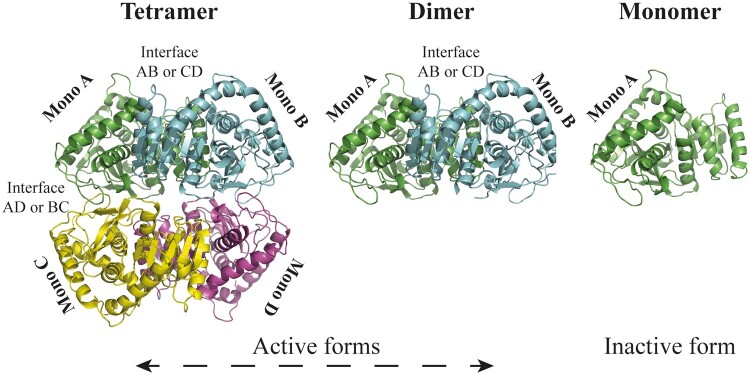
The oligomeric states of *H. mari* MalDH. The four monomers are shown in different colors. The crystal structure of the native tetrameric state is shown on the left. The central panel illustrates the crystallographic dimer associating monomers A and B. The right panel shows the crystallographic monomer. Because the dimer is a short-living form, and the monomer, an ensemble of non-homogeneous species, their structures have, so far, not been reported.

Previous AUC studies on wild type and mutants of *H. mari* MalDH have suggested an electrostatic interpretation for the KF effect. The method allows identifying the quaternary structure changes in a given solution: tetramers and/or dimers both being functional as it has been shown previously (Irimia et al. 2003; Madern and Ebel 2007). When analyzed with respect to the crystal structure of the tetrameric form (fig. 4), AUC sheds light on molecular mechanisms induced by the solvent. Below 2 M KCl (*M^f^*_½_ ∼ 1.4 M), the *H. mari* MalDH tetramer dissociates and unfolds into inactive monomers (fig. 4). Because of its higher negative charge density compared with Cl^–^, F^–^ strongly shifts the equilibrium toward lower salt concentration (KF *M^f^*_½_ 0.5 M) suggesting stronger anion binding interactions between monomer to dimer and dimer to dimer interfaces in the tetramer (Irimia et al. 2003; Madern and Ebel 2007).

In order to assess whether or not the ion charge density hypothesis is valid for other MalDHs, we recorded sedimentation coefficient values (s_20, w_) on several ancestral and modern MalDH after 24 h incubation in various concentrations of KCl and KF. The results are reported in supplementary figure S10, Supplementary Material online. Importantly, previous studies on various tetrameric MalDH have shown that protein hydration, compactness and surface properties influence experimental sedimentation coefficients, which, nevertheless, oscillate around the theoretical shape values (Irimia et al. 2003; Madern and Ebel 2007; Coquelle et al. 2010). Thus, for the sake of clarity, when close to theoretical values for active folded forms, the experimental values were replaced by explicit abbreviations in table 2, that is, (T) for tetrameric MalDH of 130 kDa with s_20, w_ =7.1S and (D) for a 65 kDa dimeric form with s_20, w_ = 4.2S. When dissociated in solution, the compact monomer of 33 kDa with an expected s_20, w_ = 2.8S is not stable, consequently, s_20, w_ values below 4.2S should be seen only as indicative of the lack of quaternary structure, corresponding rather to a molten globule state labeled (M) (Madern, Ebel, Mevarech, et al. 2000). *H. volc* MalDH is the most stable modern MalDH yet tested (from 0.5 to 3.8 M KCl) with a sedimentation coefficient s_20, w_ = 7.16 S for the active tetrameric form in 1.0 M KCl (supplementary fig. S10, Supplementary Material online). Recall that *H. mari* MalDH (KCl *M^f^*_½_ of 1.4 M) dissociates below 1.0 M KCl as shown in table 2 (Irimia et al. 2003). This indicates that the shift of *H. volc* MalDH conformational stability toward lower KCl concentration, compared with *H. mari* MalDH, results from the preservation of tetramer integrity. The s_20, w_ values at 0.2 M KCl (2.96 S) indicated that *H. volc* MalDH inactivation is concomitant with tetramer dissociation into inactive monomers (table 2, supplementary fig. S10, Supplementary Material online). Additionally, whereas *H. volc* MalDH is dissociated at 0.2 M KCl, it remains active in 0.2 M KF, displaying AUC data well fitted by a mixture of tetramers (7.2 S) and dimers (4.4 S, table 2, supplementary fig. S10, Supplementary Material online). The observation supports the electrostatic stabilization hypothesis proposed from previous measurements of *H. mari* MalDH stability in KF.*H. volc* MalDH stability in 0.2 M KF arises through the preservation of both tetrameric and dimeric active forms by F^–^ binding. Furthermore, for Anc80 MalDH, we determined s_20, w_ values of 2.2 S in 0.6 M KCl, and 4.3 and 7.4 S in 0.6 M KF. The data suggest that tetramer stabilization by the high charge density anion might therefore be an ancestral phenotype of group B and C MalDHs.

**Table 2.** Effect of a High Charge Density Anion (F^–^) on the Quaternary Assembly of Three MalDHs, from AUC Experiments in Various Salt Concentrations.

	*H. volc* MalDH		*H. mari* MalDH		Anc80 MalDH	
[Salt]	KCl	KF	KCl	KF	KCl	KF
2	—	—	**T**	**T**	**T**	—
1	**T**	—	M	—	—	—
0.6	—	—	M	**D**	M	**T/D/M**
0.2	M	**T/D**	—	—	—	—

Note.—Active tetramer, dimer, and inactive monomer are abbreviated as indicated in the main text.

The amplitude of the KF stabilizing effect (compared with KCl) varies along evolutionary trajectories (fig. 3). To the exception of Anc84 MalDH in the *H. lacusprofundii* lineage (group B) and Anc88 MalDH in the *N. magadii* lineage (group C), this phenotype appears to be shared by all the examined ancestors in groups B and C (fig. 3) suggesting a similar mode of action as revealed by AUC.

Moreover, considering that, both the LCAHa Anc78 MalDH, and *H. muko* MalDH in group A display few differences in their respective stabilizing behaviors in KCl and in KF (see *M^f^*_½_ in fig. 3), we propose that the stabilization by high charge density anions would have appeared in Anc80 MalDH. We cannot exclude that this stabilizing phenomenon pre-existed in Anc78 MalDH, and was further amplified during evolution. It was out of the scope of this study to analyze oligomeric state variation with sufficient accuracy in Anc78 MalDH, because of the small differences in salt concentration at which this effect may exist.

#### Cation Effects

**Fig. 5.**
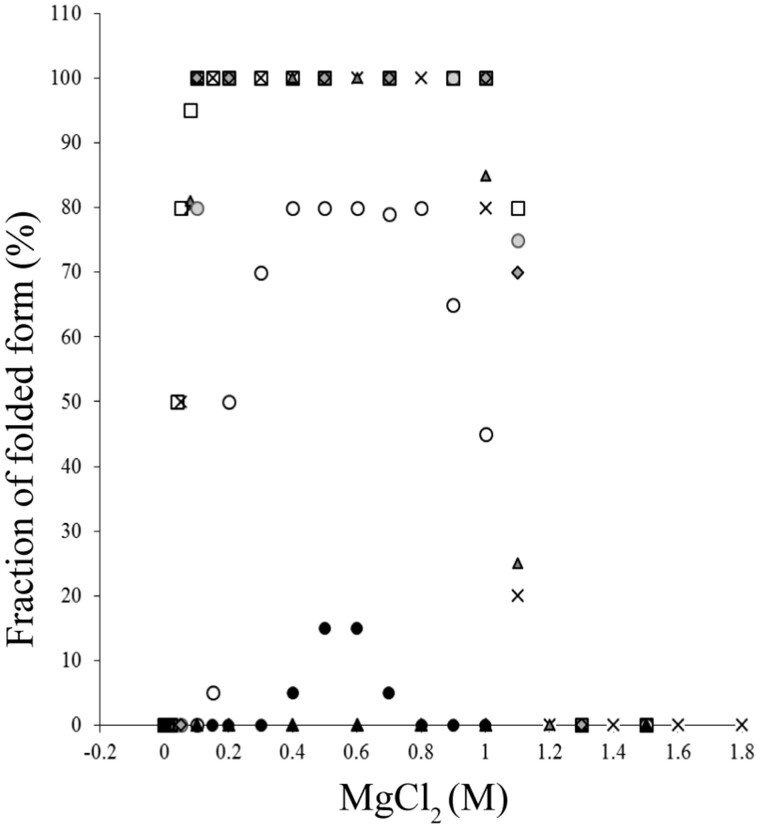
Salt-concentration dependent stability curves of various halobacterial MalDHs in MgCl_2_. Modern enzymes: *Haloarcula mukohatei* from group A (gray squares), *Haloferax volcanii* from group B (open squares), and *Natrialba magadii* from group C (black dots). Ancestral MalDHs: Anc78 (crosses), Anc80 (triangles), Anc88 (gray circles), Anc89 (open circles), and Anc90 (black triangles).

Magnesium chloride is another physiological salt that releases divalent cations of high charge density. Magnesium ions play an important role in numerous cellular mechanisms in all organisms. In Halobacteria, free Mg^2+^ intracellular concentration ranges within 0.05 M and 0.1 M (Oren 2002). Studies on the effects of MgCl_2_ on halophilic proteins are scarce, although it has been shown that divalent cations promote a favorable shift of conformational stability (i.e., *M^f^*_½_ in MgCl_2_ < *M^f^*_½_ in KCl) in *H. mari* MalDH, but also in *H. volcanii* isocitrate dehydrogenase toward lower salt concentration (Cendrin et al. 1993; Madern and Zaccai 1997; Ebel et al. 1999; Madern and Ebel 2007). The stability curves in MgCl_2_ displayed a bell shape (fig. 5), with deactivation occurring at low and high concentrations, with unfolding at the higher salt concentration due to the general effect of this chaotropic salt. We monitored whether or not such a phenomenon was detectable also in the modern and ancestral MalDHs examined in this study.

**Fig. 6.**
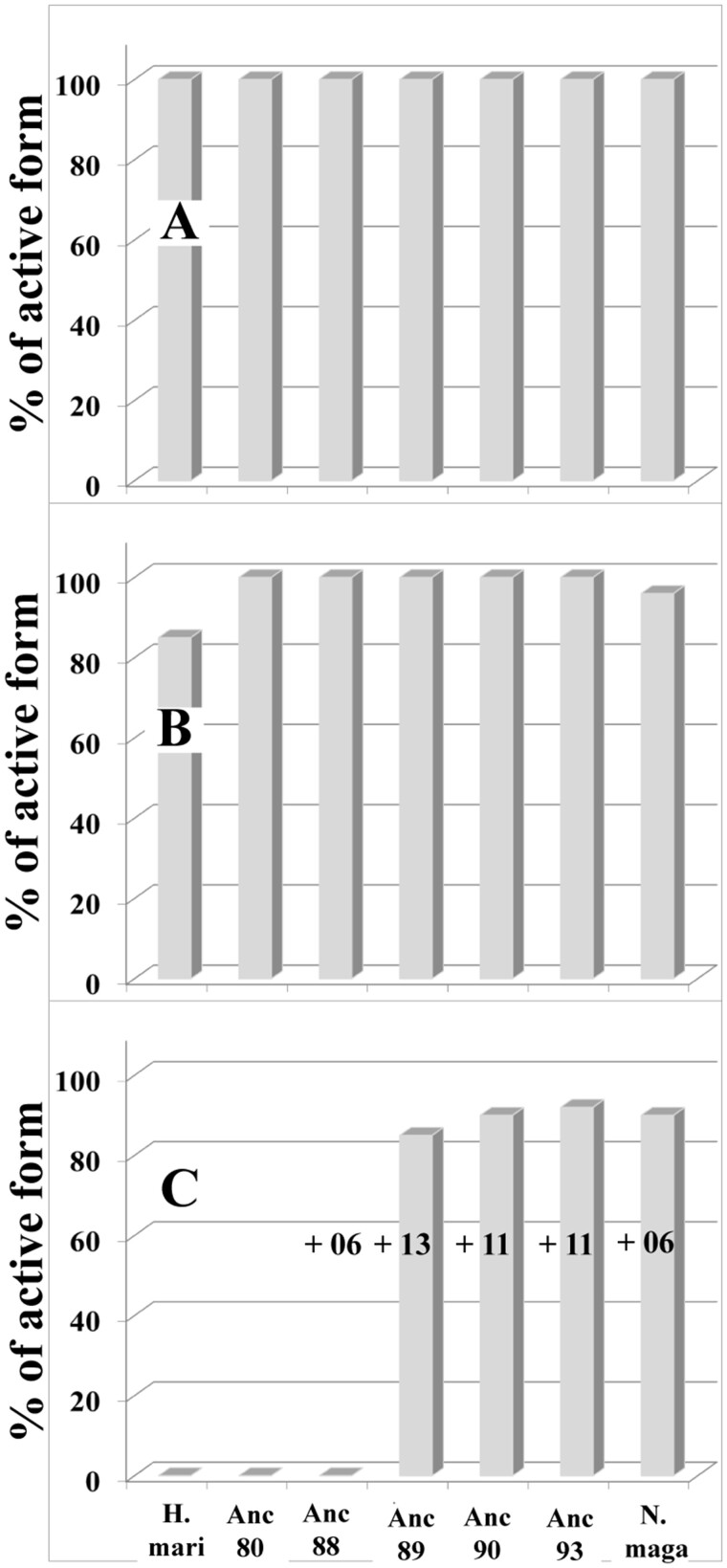
pH-dependent stability of various modern and ancestral MalDHs. Measurements were done as indicated in Materials and Methods. The data are expressed in % with respect to the values recorded prior to incubation. (*A*) pH 7, (*B*) pH 11, and (*C*) pH 12. The number of amino acid replacements accumulated in the group C MalDH, with respect to Anc80 MalDH, is indicated.

Here, we consider only the relevant change induced by MgCl_2_ on stability when its concentration decreases (i.e., below 0.6 M). Results established large differences between the studied enzymes (fig. 5). MgCl_2_ has a dramatic stabilizing effect on the ancestral Anc78, Anc80, and Anc88 MalDHs, and also on modern MalDH of *H*. *muko* (representative of group A) and *H. volc* (representative of group B). It was difficult to record a difference between *M^f^*_½_ values recorded for these five enzymes, due to the very low MgCl_2_ concentration at which the transition toward the inactive state occurs. We nevertheless estimate that *M^f^*_½_ lies below 0.1 M MgCl_2_. Our data show that the strong divalent cation stabilizing effect is an ancestral phenotype present at the root of halobacterial MalDHs Anc78. Interestingly, the curves for Anc89 and Anc90 MalDHs and *N. maga* MalDH (representative of alkaliphilic group C) in MgCl_2_ show that the divalent cation stabilizing effect is not efficient. The different values of folded fractions obtained at 0.6 M after 24 h incubation (fig. 5) suggest the dissociation and deactivation of these enzymes occurs at different rates.

To refine our understanding of MgCl_2_ stabilization effects on MalDHs, we recorded the s_20, W_ at three low KCl concentrations, supplemented (or not) by MgCl_2_ (table 3).

**Table 3.** Effect of a High Charge Density Cation (Mg^2+^) on the Quaternary Assembly of Several MalDHs, from AUC Experiments.

	*H. volc* MalDH		*N. maga* MalDH		Anc80 MalDH		Anc89 MalDH		Anc90 MalDH	
**[KCl]**	+ MgCl_2_ (M)		+ MgCl_2_ (M)		+ MgCl_2_ (M)		+ MgCl_2_ (M)		+ MgCl_2_ (M)	
	**–**	**+0.05**	**–**	**+0.50**	**–**	**+0.05**	**+0.05**	**+0.5**		**+0.50**
0.6	—	—	—	—	—	—	*M*	**T**	—	*M*
0.2	*M*	—	—	—	—	—	—	—	—	—
0.1	—	**T**	—	*M*	—	**T**	—	—	—	—

Note.—Active tetramer, dimer and inactive monomer are abbreviated as indicated previously.

Recall that in molar concentration of KCl, Anc80, Anc89, Anc90 and *H. volc* MalDH, display s_20, W_ values in agreement with the presence of the tetrameric state (supplementary fig. S10, Supplementary Material online). As discussed, in 0.1 M KCl (supplementary fig. S5, Supplementary Material online), all the enzymes are dissociated and inactive. On one hand, when the 0.1 M KCl solution is supplemented with 0.050 M MgCl_2_, Anc80 and *H. volc* MalDH behave as tetramers (table 3 and supplementary fig. S10, Supplementary Material online). On the other hand, within group C, Anc90 MalDH and *N. maga* MalDH sedimentation profiles at 0.6 and 0.1 M KCl, respectively, plus 0.5 M MgCl_2_, were well fitted by a population of inactive monomers, consistent with the stability profile measured in MgCl_2_ (fig. 5). When 0.5 M MgCl_2_ is added to the 0.6 M KCl solution, Anc89 MalDH is mainly tetrameric with s_20, W_ = 7.4 S. At lower concentration of MgCl_2_ (0.05 M), the Anc89 MalDH s_20, W_ value of 1.9 S shows that the enzyme is dissociated into monomers. The various oligomerization states induced by different concentration of MgCl_2_ likely reflect subtle changes in cation binding affinities between enzymes. It is out the scope of this work, however, to analyze this phenomenon in more detail. Our data demonstrate that, when it is considered as beneficial for MalDH stability, the effect of Mg^2+^ is due to its capacity to maintain the integrity of the active tetrameric state, even at very low KCl concentration. Importantly, as indicated by the absence of 4.2 S peaks in AUC (supplementary fig. S10, Supplementary Material online), dimeric active states are not stabilized by MgCl_2_, in contrast to anions of high charge density. This suggests that anion and cation binding sites exert different controls on monomer–monomer and dimer–dimer interfaces.

Altogether, our data suggest that the capacity to stay folded even at low KCl concentration in the presence of a secondary physiological salt, such as MgCl_2_ is an ancestral phenotype of halobacterial MalDH, possibly lost in group C MalDH. It should be considered as beneficial for halophilic lifestyle adaptation (see Discussion) because it has been conserved in lineages of group A and B. How this phenotype has been strongly weakened in group C, is addressed by a series of complementary investigations presented below.

### The Halo-Alkaliphilic Phenotype of MalDH Is a Secondary Evolutionary Trait

Haloalkaliphilic Archaea are able to grow both in high salt concentration and high pH up to 11, with a matching cytoplasmic pH value (Falb et al. 2005). High pH is disruptive of electrostatic interactions and has a strong deleterious effect on protein stability (Dill 1990). We tested whether or not MalDH isolated from the haloalkaliphilic Archaea *N. magadii* and reconstructed ancestral enzymes in group C displayed specific features that may counterbalance this effect (Fig. 6). MalDH from *H. marismortui* and ancestors Anc80 and Anc88 MalDHs were considered for comparison. We incubated the enzymes for 24 h in 2.0 M KCl solutions buffered at different pH values ranging from pH 6 to pH 13, and monitored stability by using residual activity measurements. Anc80 and Anc88 and *H. mari* MalDH were unstable at pH 12, unlike Anc89, Anc90 and Anc93 and *N. maga* MalDH, which remained stable. Because of chemical incompatibility of high pH with sapphire optical cells, AUC data were not recorded. Consequently, we could not determine as previously to what extent the favorable stability changes results from equilibrium shifts between oligomers and higher. Nevertheless, data obtained with the Anc89, Anc90, Anc93 and *N. maga* MalDH at pH 12 suggest that some replacements were selected in Anc89 MalDH and conserved in the *N. maga* lineage, likely providing some beneficial effect in high pH environments. Intriguingly, the stability properties of group C MalDH, in high pH occurred concomitantly with the disappearance of the stabilizing effect of MgCl_2_, (see above). This observation is in agreement with the fundamental process of protein evolution, which has established that trade-offs between properties are responsible for the emergence of new phenotypes (Tokuriki et al. 2008).

We analyzed the amino acid replacement pattern of MalDH within group C (supplementary fig. S8, Supplementary Material online). From Anc88 to Anc89 MalDH, 13 replacements are observed. When their position is analyzed with respect to the crystal structure of *H. mari* MalDH (PDB code 2J5K), the replacement that occurred at position 239 (H–R) suggests its importance with respect to stability. Indeed, in contrast to other 12 positions, at position 239 the side chains of H239 in *H. mari* MalDH face each other across the interface in the dimer of active dimers (AB and CD) that makes up the tetrameric assembly. At this particular position, the replacement of an amino acid (H) with a pKa that is sensitive to pH variation, by R that is not, would have been a favorable replacement.

### Evolution of Enzymatic Activity

**Fig. 7.**
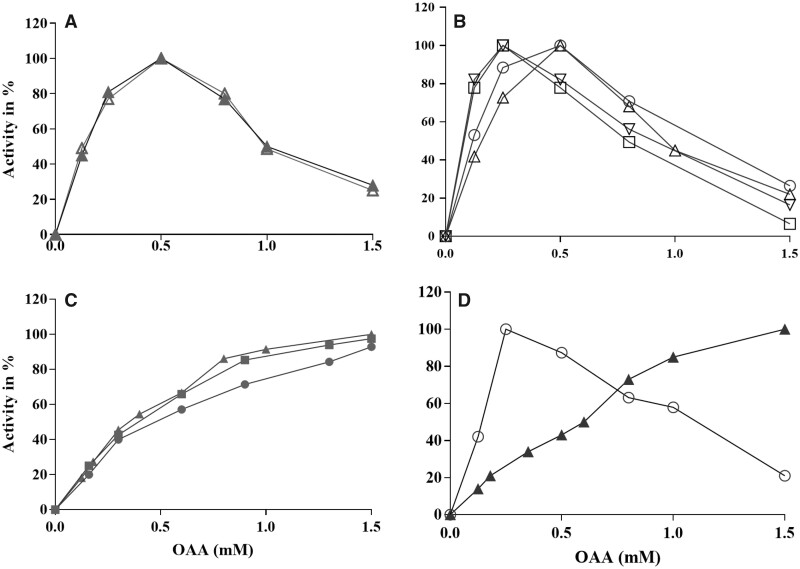
Enzymatic activity profiles of ancestral and modern MalDHs from different groups. The results are expressed in % of maximal activity. (*A*) LCAHa Anc78 MalDH (closed triangles) and Anc80 MalDH (open triangles). (*B*) Anc81 MalDH (open circle), Anc84 MalDH (open square), *H. volc* MalDH (open triangle), and *H. lacu* MalDH (inverted triangle). (*C*) Anc82 MalDH (gray triangle), and Anc88 MalDHs (gray square) and *N. maga* MalDH (gray dots). (*D*) *H. muko* MalDH (open circle) and *H. mari* MalDH (black triangle).

Finally, we explored the enzymatic properties of MalDHs along the evolutionary pathways. We monitored the enzymatic activity at various concentration of their substrate (oxaloacetate, OAA) at KCl concentration of 3.8 M. Because exhaustive measurements of the steady state parameters of these enzymes were out of the scope of this study, we simply present the results in a comparative mode allowing to see the relative effect expressed as % of the maximal activity observed. The unexpected results indicate that MalDHs split into two groups, which differ by their capacity to be inhibited or not by increasing concentration of OAA (fig. 7). Activity profiles for both the deepest ancestors Anc78 and Anc80 MalDHs show a typical phenomenon of inhibition by an excess of substrate (fig. 7*A*). This effect can be, therefore considered as an ancestral feature in halobacterial MalDHs. In group B MalDHs, the maximal activity is reached with 0.4 mM of OAA; at higher concentration, the activity of all the enzymes is inhibited (fig. 7*B*), indicating that this property was conserved in this group. In contrast, in group C as well as in the ancestor of group C and *H. mari* MalDH (Anc88), the inhibition by high concentration of OAA is abolished (fig. 7*C*). In group A, the activity profiles of *H. muko* and *H. mari* MalDHs are completely different (fig. 7*D*). *H. muko* MalDH is representative of group A, its activity profile shows an inhibitory effect at OAA concentration higher than 0.5 mM. Because this is the ancestral behavior measured for the root MalDH Anc78, it could be seen as an inherited property shared by enzymes from group A. Our measurements showed that *H. mari* MalDH is not sensitive to inhibition by high concentration of OAA (fig. 7*D*), an observation in agreement with a previous work on this enzyme (Hecht et al. 1989). The peculiar behavior of *H. mari* MalDH is therefore consistent with an HGT from a lineage related to the group C ancestor. RP effects that explain the change in enzymatic activity of halobacterial MalDHs, is under investigation using site-directed mutagenesis.

### An Intermediate Ancestral Halobacterial MalDH with Nonhalophilic-Like Properties

The unexpected stability down to 0.05 M KCl of Anc84 MalDH (from group B), in the lineage leading to *H. lacu* MalDH, was carefully analyzed. With an *M^f^*_½_ value lower than 0.1 M KCl (see fig. 2), the enzyme is significantly more stable in low salt concentration than all other considered halobacterial MalDHs.

**Fig. 8.**
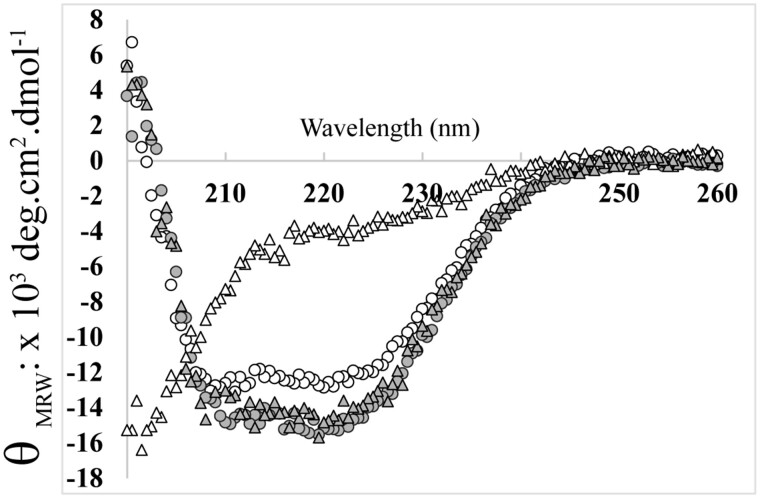
Circular dichroism signal of Anc84 MalDH (circles) and *H. lacu* MaDH (triangles), after incubation in 0.06 M (white) or 1.0 M (gray) KCl.

In 1.0 M KCl, the CD spectra of Anc84 MalDH and *H. lacu* MalDH display strong negative values of molar ellipticity at 210 nm and 222 nm, and positive values below 200 nm (fig. 8), typical of folded alpha + beta proteins, similar to those observed previously for *H. mari* MalDH at 4 M KCl (Madern, Ebel, Mevarech, et al. 2000). A similar spectrum indicates that Anc84 MalDH is still folded in 0.06 M KCl. In contrast, at the same low KCl concentration, *H. lacu* MalDH displays a CD spectrum typical of an unfolded protein. We tested that the Anc84 MalDH unfolds after dialysis in water as it is expected with a halophilic enzyme. The Anc84 MalDH behavior was further examined by monitoring its sedimentation coefficient (supplementary fig. S11, Supplementary Material online). As expected, at 1.0 M KCl, the s_20, W_ value of 7.6 S indicates the presence of tetramer (table 4).In both 0.06 M and 0.1 M KCl, Anc84 MalDH forms dimers, displaying s_20, W_ values of 4.8 S and 4.9 S. Surprisingly, the s_20, W_ value in 0.1 M KF indicates that the Anc84 MalDH still forms a dimer, whereas with other MalDH, we always obtained stabilization of the tetrameric state (see previous section). In the presence of 0.05 M MgCl_2_, s_20, W_ values are in agreement with the tetrameric state. Our data suggest, therefore, that the peculiar behavior of Anc84 MalDH results from beneficial replacements acting at the AD and BC interfaces (see fig. 4), which promote stronger binding between the active dimeric units AB and DC to make the tetramer, even at low salt concentration.

**Table 4.** Oligomeric State Variation of Anc84 MalDH in Various Salt Conditions.

	Anc84 MalDH			
[Salt]	KCl		KF	KCl
				+ 0.05 M MgCl_2_
1.0	**T**	—		—
0.1	**D**	**D**		**T**
0.06	**D**	—		—
0.00	—	—		**T**

Note.—Abbreviations are as indicated previously. Data in the left columns help to see the putative effect of anions of high charge density. Columns on the right reflect the effect of divalent cation. The measurement done at low MgCl_2_ concentration in absence of KCl was done after dialysis of the sample.

## Discussion

### Salt-Dependent Stability of Halophilic MalDH Could Not Be Used as a Salinity Sensor

Understanding the evolution of organisms relies on the capacity to understand the processes that shaped cellular components under environmental selective pressures. Such questions may be appropriately addressed by using ASR approaches. Along these lines, several studies based on ASR support the hypothesis that, during early stages of prokaryotic evolution, high environmental temperature was a major determinant shaping the proteomes (Boussau et al. 2008; Groussin and Gouy 2011). In the framework of such a scenario, resurrection of several ancestral proteins from various bacterial and archaeal lineages revealed high resistance against thermal unfolding (Gaucher et al. 2003; Perez-Jimenez et al. 2011; Hobbs et al. 2012, Risso et al. 2013; Romero-Romero et al. 2016).These results are based on the reasonable assumption that the conformational stability of each protein is related directly to the environmental temperature of its host and could therefore be used as a molecular thermometer (Gaucher et al. 2003; Hobbs et al. 2012). Moreover, such a conclusion applied to the average paleo-environmental temperatures at the scale of the whole planet. Hence, in the case of temperature, application of ASR to the estimation of paleo-environmental conditions is possible because the conditions were global and effects on proteins, direct. Likewise, could the ASR and resurrection of ancestral halobacterial MalDHs achieved in our work help to infer the salt composition of ancient environments? Several limitations, in our opinion, unfortunately preclude direct interpretation of the estimated ancestral MalDH phenotypes as adaptations to paleo-environmental salt concentrations.

First, we found no correlation between the salt-concentration dependent stability of cytoplasmic MalDH from modern species (as indicated by *M^f^*_½_ values, table 1) and the NaCl concentration of their natural habitats. Furthermore, recent studies have shown that the salt content of halobacterial cytoplasm cannot be described simply as containing a high KCl concentration. It corresponds rather to a complex ion mixture regulated by the nature, the number, and relative efficiency of membrane transporters for various ions and compatible solutes (Becker et al. 2014). In their natural environments, halobacterial species permanently face important variations in salinity due to rainfall and evaporation. In order to compensate for unfavorable variations in osmotic pressure, salt type and concentration within the cytoplasm can be transiently decorrelated from environmental fluctuations. Consequently, it is not possible to infer the salt composition of the paleo-environment that prevailed during the evolution of Halobacteria by using the ancestral MalDH conformational stability as a salinity sensor.

We assume that, in a near future, such estimates could rely on the examination of closely related halobacterial species and their genomic contents, including ion transporters and proteins regulating cytoplasmic ion content. Especially, since Halobacteria are thought to have diverged from methanogenic Archaea (Forterre et al. 2002). A recent publication has shown that the methanogen-to-halophile transition, in Archaea, has involved a new intermediate group of organisms called Hikarchaeia (Martijn et al. 2020). Experimental characterization of their enzymes would increase our understanding of halophilic adaptation.

### Molecular Evolution in Halobacteria, a Case Study

**Fig. 9.**
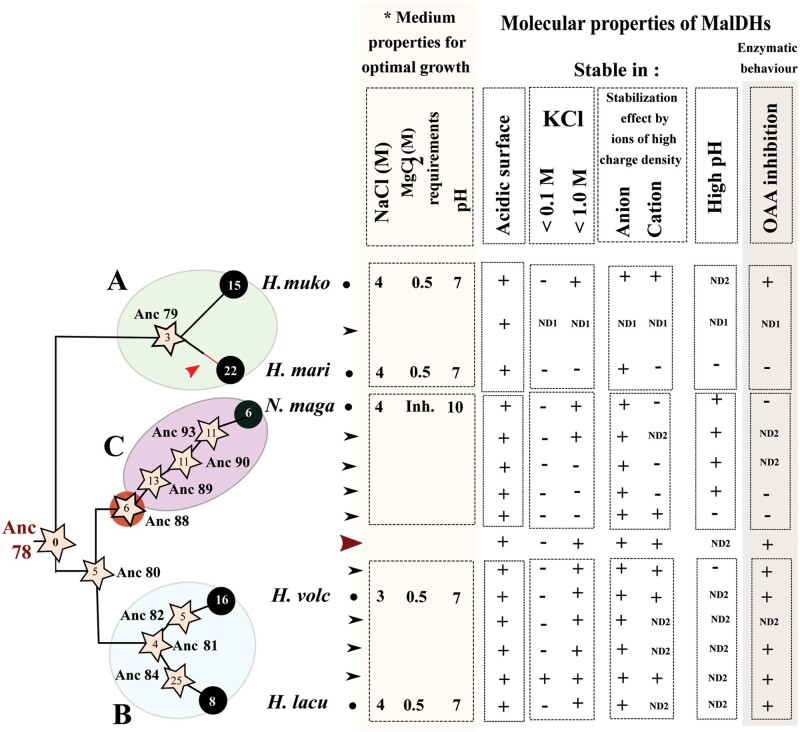
Summary of main results about halobacterial MalDH evolution. Left: schematic phylogenetic relationships among MalDH within Halobacteria. *A*–*C* refer to the various groups. Stars and circles correspond ancestral or modern MalDHs, respectively. The number of amino acid replacements accumulated since LCAHa Anc78 MalDH is indicated within each enzyme. Molecular properties of MalDHs: observed (+), absent (–), or not determined (ND). ND1 refers to refolding problems and ND2 to not done or failed experiments. Data on *H. mari* MalDH are obtained from Ebel et al. (1999) and Madern and Zaccai (1997). (*) Data obtained from halohandbook: https://haloarchaea.com/halohandbook/.

We have, for the first time, experimentally discriminated at the molecular level how modern enzymes from extremophilic organisms have acquired their properties either through the modification of inherited genetic information (RP, genetic drift and Darwinian selection) or through non-genealogical acquisition of genetic material by HGT. We used halophilic Archaea as appropriate system models, in part, because they have a well-documented capacity for generating genetic variation through HGT (Ram-Mohan et al. 2014 refs therein). Our main results describing successive phenomena that shaped the adaptation of halobacterial MalDHs facing extreme salt concentrations are summarized in figure 9.

We examined the effects of RP on three different molecular properties of MalDHs. In particular, we tried to see whether solubility, conformational stability and enzymatic activity evolved concomitantly in order to fit the peculiar salt conditions encountered in the cytoplasm of modern cells.

#### Solubility

Acidic amino acid enrichment is a well-established structural feature of modern halophilic proteins that help them to stay soluble at high salt concentration (Coquelle et al. 2010, Talon et al. 2014). We showed that the strong acidic surface enrichment of halobacterial MalDH is a conserved ancestral phenotype, shared since the last common ancestor of the studied lineages. However, an acidic composition does not indicate a moderate or extreme halophilic lifestyle. Indeed, some studies using halophilic bacterial species have shown that a strict relationship between a highly acidic proteome and high salt in the environment does not hold (Deole et al. 2013; Oren 2013). Furthermore, even if acidic enrichment is a phenotype that favors solubility at high salt concentration, experimental studies have shown it is also beneficial at low salt concentration (Coquelle et al. 2010). Finally, previous attempts to classify pI variations (at the proteomic level) to different ecological classifications showed that extreme and moderate halophilic organisms could not be distinguished (Kiraga et al. 2007). These observations indicated that global acidic enrichment in a protein becomes favorable above a certain (low) threshold of salt concentration, and provides an important adaptive advantage in high environmental salt concentrations. Consequently, the use of protein acidity values as a proxy failed to predict with accuracy ancient environmental conditions in which the LCA of extant Halobacteria lived. One can only conclude that this shared ancestral phenotype was advantageous and remained conserved during the adaptation of modern species within Halobacteria to moderate or extreme halophilic lifestyles.

#### Conformational Stability

Theoretical and experimental studies indicated that evolutionary trajectories of enzyme oscillate between increasing and decreasing stability phenotype drifts (Hart et al. 2014; and reference therein). Along this line, monitored KCl *M^f^*_½_ values revealed that MalDH conformational stabilities increase and decrease independently, with distinct amplitudes, during the evolution of the three analyzed lineages down to modern MalDH. The monitoring of oligomeric states revealed that this change in stability is mainly due to dissociation of the active tetrameric assembly into inactive monomers. We also found that anions of high charge density, such as fluoride, shift protein stability, in most of cases, toward lower salt concentration than chloride. In two alkaliphilic ancestral MalDHs (Anc89 and Anc90) and *H. mari* MalDH this ionic effect is strongly stabilizing with a *M^f^*_½_ decrease of ∼1 M. This results in the capacity of these MalDHs to behave also as active dimers in the presence of strongly stabilizing anions. Numerous biophysical and structural investigations using the *H. mari* MalDHs strongly support the existence of weak and strong anion and cation binding sites that contribute to maintain the quaternary assembly (Madern and Zaccai 1997; Ebel et al. 1999; Madern and Ebel 2007). Our data demonstrate that when the salt concentration decreases to a given threshold, the tetrameric state of the various considered ancestral and modern MalDHs dissociates, triggering concomitant deactivation and unfolding. The KCl, CsCl and KF *M^f^*_½_ variations along the evolutionary trajectories can be, therefore, well explained by affinity changes of ion-binding sites due to direct and indirect effects of amino acid replacements. Some of which are strongly beneficial, by lowering the rate of dissociation between oligomers when the salt concentration decreases. Although the various considered MalDHs behave differently according to salt type and concentration, the ion binding site mechanisms appear to be shared by all the halobacterial MalDH since the LCAHa. Additionally to the shared acidic surface enrichment, this ion-binding site phenotype might be interpreted as resulting from an adaptation of the LCAHa to a salted environment.

We observed that the stabilizing effect of cations of high charge density is effective on the two oldest considered LCA MalDHs (LCAHa: Anc78, and LCA of group B + C: Anc80) as well as on the modern MalDHs of *H. volc* and *H. muko*, belonging to species group A and B (fig. 8). These enzymes, at very low KCl concentration, in the presence of 100 mM MgCl_2_, can be stabilized only as tetramers. The data strongly suggest that 1) cation-binding sites at the interface between the dimer of active dimers (AB and CD) contribute to tetramer assembly, 2) Mg^2+^ may occupy these cation-binding sites with a higher efficiency than K^+^. Interestingly, we showed that Anc84 MalDH still forms a tetramer in pure solution of 0.05 M MgCl_2_. This suggests that amino acid replacements in its primary sequence have dramatically increased its binding affinity for Mg^2+^ through mechanisms that remain to be elucidated.

We noted that the stabilizing effect of Mg^2+^ is effective only on MalDHs from group A and group B. These are representative of halobacterial species living in salty environments at neutral pH, which do not show the loss of stabilization by Mg^2+^ occurring in the MalDH of the haloalkaliphilic group C LCA (Anc89), as well as in the haloalkaliphilic *N. maga* MalDH. Additionally, we showed that all modern and ancestral alkaliphilic MalDH acquired stabilization properties at high pH (fig. 8). This phenomenon likely relies on the acquisition of an amino acid (R239) in a structural key position, for which the ionization state and therefore the capacity to establish interactions is not abolished at high pH values. We noticed that this replacement occurred when the evolutionary trajectory is the most destabilizing with respect to the KCl requirement (KCl *M^f^*_½_ of Anc89 MalDH = 1.4 M). This observation is in agreement with studies showing that, during an evolutionary process, protein stability changes do favor the acquisition of new properties (Tokuriki et al. 2008). Thus, even if the variation of MalDH conformational stability does not directly reflect variations of environmental salt concentrations, the identified drastic changes in stability phenotypes suggest an adaptation to haloalkaliphilic conditions in the LCA of Natrialbales order (Group C).

*H. mari* MalDH is unstable in MgCl_2_. It does not behave, however, as an alkaliphilic MalDH (this work and Madern and Zaccai 2007). Its primary sequence shows a histidine residue at position 239, implying that the decrease of Mg^2+^binding capacity of *H. mari* MalDH might be a convergent phenotype resulting from amino acid replacements occurring after it diverged from the haloalkaliphilic group C MalDHs (Anc88, see fig. 8).

Amino acid replacements do not only exert a local effect but also effects that propagate through the dynamical properties of the enzyme (Bigman and Levy 2018; Yu and Dalby 2018). Diverging amino acid replacements may have influenced the phenotypic properties of *H. mari* MalDH through long-range distance effects rather than specifically at the interface between dimers of active dimers. The ongoing description of a halophilic MalDH crystal structure with its associated solvation shell would provide the relevant information to describe such effects in detail.

#### Enzymatic Activity

According to the traditional comparison of properties of proteins isolated from extant organisms, it is commonly admitted that a stability–activity tradeoff drives adaptive evolution of enzymes (Somero 2004; Studer et al. 2014) even if this has been challenged by Nguyen et al. (2013). From previous comparative studies with its nonhalophilic homologs, it has been commonly admitted that the necessary flexibility of *H. mari* MalDH, required to function at high salt concentration, was the consequence of a compromise which has decreased its stability at low salt concentration. Latter, it was shown that a clear conclusion concerning the salt-concentration dependency of activity could not be established easily because most of the measurements have been done without taking into account the strong salt-concentration dependency of affinity for the substrate (Hecht et al. 1989; Madern et al. 2004). We therefore changed the strategy of analysis, by recording the substrate-concentration dependency of activity at a unique KCl concentration of 3.8 M for all the MalDHs under study. Thanks to this methodology, we discovered that ancestral and modern MalDHs display two kinds of phenotypes: some are sensitive to inhibition by high substrate concentration during their catalytic cycle; others are not (i.e., *H. mari* MalDH, alkaliphilic group C MalDH and their common MalDH ancestors). Our unexpected findings showed that oldest considered MalDH LCAs (LCAHa: Anc78, LCA of group B + C: Anc80), which are inhibited by high OAA substrate concentration, diverged into a non-inhibited enzyme in the ancestor of *H. mari* MalDH and of the alkaliphilic clade C MalDH (Anc88, see fig. 8). Interestingly, we analyzed that none of the amino acid replacement concerns those involved in substrate recognition and catalysis within the catalytic site (not shown), suggesting long range effects through change in protein dynamics, such as it has been observed in others enzymes (Zoi et al. 2016). Interestingly, this non-inhibited phenotype, appeared in a lineage related to the alkaliphilic halobacterial (group C) ancestor, is retained in the *H. mari* (group A) MalDH. This further demonstrates that *H. mari* acquired its MalDH through HGT and replacement of its inherited copy during evolution. In a recent work on the response of ADP-dependent kinases of *Methanosarcinales* facing moderate salt concentration, it has been shown that non-inhibited modern orthologs evolved from an inhibited ancestral state (Gonzales-Ordenes et al. 2018). We propose that the selection of enzymes less sensitive to inhibitory effects on their activity is one of the selective advantages that may contribute to adaptation to salty environments.

## Conclusion: The Evolution of Halophilic Proteins

We are now able to propose a detailed scenario for the evolution of a halophilic enzyme, based on our biochemical, structural and evolutionary analyses.

Halobacteria are generally considered to be “salt-in” strategists—actively accumulating potassium and chloride ions to maintain an isosmotic cytoplasm in order to prevent loss of water to the hypersaline environment. Because it has been studied extensively, MalDH from *H. marismortui* has been considered for a long time to be the best model for a halophilic enzyme. In particular, a solvation shell of hydrated ions coordinated by carboxyl groups on the protein surface has been proposed to maintain *H. mari* MalDH stable and soluble at high salt (Madern et al. 2000) with a consequence that the structure would unfold already if the salt concentration fell to relatively high value (2 M KCl). By taking into account a large set of ancestral and modern orthologous halobacterial MalDHs, our study challenged the general validity of this model. We showed that *H. mari* MalDH instability at a relatively “high” salt concentration (2 M KCl) is a specific evolutionary fate, possibly inherited through an ancestral HGT, rather than the general behavior of halophilic enzymes. In contrast, most halobacterial MalDHs, as well as their oldest common ancestors, appear as moderate halophilic enzymes with a better tolerance for lower cytoplasmic salt concentration than observed with *H. mari* MalDH. In turn, within Halobacteria, some species, such as *H. marismortui* may have subsequently adapted to obligate extreme halophilic lifestyles, with possible loss of tolerance to cytoplasmic salt depletion. Moreover, our study did not show any evidence concerning a gradual shift in acidic amino acid composition. Because this surface modification is strongly beneficial to favor solubility at high salt concentration, the property was fixed over time. We found that most of ancestral and modern MalDHs could maintain their conformational stability at lower KCl concentration, especially in the presence of another physiological salt, such as MgCl_2_. This is in agreement with an intracellular measurement reporting an intracellular content of 100 mM MgCl_2_ in a halobacterial species (Matheson et al. 1976), and with genomics studies revealing the presence of several types of Mg^2+^ transporters such MgtE and CorA-like membrane proteins (Becker et al. 2014). We found that anions of high change density were also strongly efficient with respect to stability. Our data, which account for the complexity of the salt composition within the cytoplasm of Halobacteria, showed that the conformational stability of halophilic proteins varied over evolution. We propose that the capacity of a halophilic protein to stay folded even at low salt concentration may, therefore, be an efficient ancestral mechanism to survive osmotic down shock. Biophysical models of protein evolution explain that selection attempts to maintain the existing structure, function and dynamical properties when these remain beneficial in a new environment (DePristo et al. 2005; Tokuriki and Tawfik 2009b). In this framework, most of the amino acid replacements occurring during the evolution of MalDH in groups A and B would have nearly neutral effects with respect to stability.

The role of stabilizing versus destabilizing mutations, in the evolution of protein properties, is controversial (Bloom et al. 2006; Merski and Shoichet 2012, Bigman and Levy 2020). In the case of destabilizing mutations, their detrimental effects are buffered by the presence of molecular chaperones, so that they may promote evolvability (Geller et al. 2018). Here, we observed that an accumulation of destabilizing replacements, such as observed in group C MalDH ancestors, has rendered the enzyme prone to evolve new properties. Indeed, we found an unexpected molecular tradeoff in haloalkaliphilic group C showing that the selection of new enzymatic properties (i.e., nonsubstrate inhibited MalDH) and the loss of the capacity to enhance conformational stability at low salt by Mg^2+^ are concomitant with the adaptation to high pH environments. This evolutionary trait makes sense with respect to the specific chemical constraints encountered in alkaliphilic environments. Indeed, because of the high pH encountered in alkaliphilic environments divalent cations mainly exist as insoluble carbonates, consequently Ca^2+^ and Mg^2+^ availability is very low. In addition, MgCl_2_ inhibits cellular growth of species from the *Natrialba* genus (Tindall et al. 1980). When deprotonated malate and oxaloacetate exist, as reactive divalent anions, they may easily react with divalent cations, such as Ca^2+^ or Mg^2+^ (Butler 1998).

Because of this chemistry, one may reasonably consider that the concentration of oxaloacetate is higher in a haloalkaliphilic cytoplasm depleted in divalent cations than encountered in other halobacterial species; a situation which would have been less favorable for the functionality of an OAA-inhibited MalDH. We propose that the selection of MalDHs nonsensitive to substrate inhibition was advantageous during the expansion of species within Halobacteria to fit new environmental conditions, by preventing a decrease of metabolism efficiency.

## Materials and Methods

### Main Phylogenetic Reconstruction Steps

Several phylogenetic reconstruction steps are performed in order to obtain the ancestral MalDH sequences. First, a phylogenomics approach is used to estimate a species tree. Genes are gathered (section Reconstruction of gene families) and a species tree is reconstructed using ML methods (section Reconstruction of the species tree). Second, a collection of MalDH trees is computed using Bayesian methods (section Reconstruction of the MalDH sequence-only tree). Third the latter trees are reconciled with the species tree using a ML approach (section Species tree-gene tree reconciliation). Fourth, using the reconciled MalDH joint tree, MalDH sequence are aligned anew accounting for indel events and ancestral MalDH sequences are estimated using a Bayesian method (section ASR of MalDH). At each steps, control experiments are performed using ML or Bayesian models.

### Reconstruction of Gene Families

From the collection of 51 proteomes, homologous gene families have been obtained with an all-against-all BLAST approach. The program Silix was then used to cluster protein sequences into homologous families (Miele et al. 2011). Default parameters were considered for the clustering: sequences having >30% similarity and >80% coverage were clustered together into a homologous gene family. Unicopy gene families having >80% of taxonomic coverage were conserved, these 240 gene families were further aligned with Prank (Löytynoja and Goldman 2005; Löytynoja and Goldman 2008), internally used in Guidance (Penn et al. 2010) to trim ambiguously aligned sites by taking into account the uncertainty of the guide tree during the alignment procedure. Ambiguously aligned sites were further trimmed with Gblocks (Castresana 2000), with standard options and with gaps allowed. Finally, the bppSeqMan program belonging to the bppSuite of programs (Duteil and Boussau 2008) was used to eliminate sites containing >20% of gaps.

### Reconstruction of the Species Tree

The species tree has been inferred with a concatenation approach. After the concatenation of the 240 gene families, the final alignment contains 50,135 amino acid positions. Different strategies were employed to reconstruct the species tree. The LG replacement model (Le and Gascuel 2008) and a Gamma distribution with four categories were used in PhyML (Guindon et al. 2010) to reconstruct the tree with 100 bootstraps. The LG model assumes that the evolutionary process is constant between lineages and across sites. The CAT and CAT+GTR models (Lartillot and Philippe 2004) implemented in Phylobayes (version 3.3, Lartillot et al. 2009) were also employed. CAT and CAT+GTR assume that the process is heterogeneous among sites and constant between lineages. We computed 1,000,000 cycle long MCMC chains, saving a sample each 10 cycles. The 1,000 first samples were discarded as burnin. Two independent chains were executed for each experiment. The chain's convergence was assessed if the difference of posterior log likelihood across MCMC chains is less than three times the estimated standard error and the tree bipartition PP differ by <0.1. Finally, the COaLA replacement model was also used with bppML (Duteil and Boussau 2008) COaLA is site-homogeneous but implements a time-heterogeneous model that allows to model the variation of global compositions between lineages. As topology exploration is not feasible with time-heterogeneous models in bppML, COaLA was used to test alternative topologies regarding the position of the root of the Halobacteria clade. To discriminate between alternative topologies, AU tests were performed with Consel (Shimodaira and Hasegawa 2001).

To improve the resolution of the phylogeny of Halobacteria, and especially the position of its root, an elimination of the fast-evolving positions was realized to reduce systematic errors (Brinkmann et al. 2005; Philippe et al. 2005). We used the site-specific posterior rates computed by PhyML with the use of the Γ distribution to remove gradually the fast-evolving sites (by fractions of 10% of sites).

### Reconstruction of the MalDH Sequence-Only Tree

The MalDH gene family was reconstructed with Silix (Miele et al. 2011). The 51 MalDH sequences were then aligned with Muscle (Edgar 2004) used in Guidance (Penn et al. 2010), which allows to trim ambiguously aligned sites owing to uncertainty in the guide tree topology. Remaining ambiguously aligned sites were removed with Gblocks (Castresana 2000). The Bayesian sequence-only tree was reconstructed using the CAT+GTR model implemented in PhyloBayes 3.3 (Lartillot et al. 2009). We ran 1,000,000 long independent MCMC chains, saving a sample each 10 cycles, and we discarded the 1,000 first samples as burnin. The chain's convergence was assessed if the tree bipartition PP differ by <0.05*.* The consensus sequence-only tree was then reconciled with the species tree.

### Species Tree-Gene Tree Reconciliation

A prereleased version of the Amalgamated Likelihood Estimation (ALE) algorithm (ALE v0.1) (Szöllosi, Tannier, et al. 2013) was used to compute the ML MalDH joint tree. ALE uses the model described in (Szöllosi, Rosikiewicz, et al. 2013) to search for the best scenario of duplications, transfers and losses of genes and efficiently explores the space of joint trees that maximize the joint likelihood between the sequence and reconciliation information. ALE requires a time calibrated species tree to compute ML estimates of transfer rates. Divergence times were estimated using PhyloBayes 3.3 from the genomic concatenation having 50% of its fastest evolving sites filtered out (see above).

We used the CAT replacement model, the Log Normal relaxed molecular clock, no calibration point, and a flat root prior defined by a 1 billion years expectation and standard deviation. The species tree was rooted with the *Thermoplasmatales*. The MCMC chains were elongated for 1,000,000 cycles, a sample was saved every 10 cycles, and 1,000 first samples were discarded as burnin. Convergence is estimated to be reached when the difference of posterior log likelihood across MCMC chains is less than three times the estimated standard error. ALE also uses a sample of gene trees to compute conditional clade probabilities (Höhna and Drummond 2012), which can be used to approximately estimate the PP of a gene tree that can be amalgamated from clades present in the sample. The sample of posterior trees computed by PhyloBayes 3.3 on the MalDH alignment (see above) was provided to ALE.

### ASR of MalDH

The MalDH joint tree was used as a final guide tree to Prank, which is very sensitive to the choice of the guide tree (Löytynoja and Goldman 2005). The Prank alignment and the joint tree were used to compute the ancestral sequences. Moreover, one of the key outputs of Prank is the inferred history of the insertion/deletion events leading to the extant gap pattern. This history is used to filter out from the computed ancestral sequences all ancestral sites that are inferred as gaps by Prank.

To reconstruct ancestral MalDH sequences in ML, the bppAncestor program (Dutheil and Boussau 2008) was used with the marginal reconstruction approach (Yang et al. 1995). For a given node at a given site, bppAncestor makes use of the ML estimates of branch lengths and model parameters obtained with bppML to compute the PP of each possible ancestral states. The state having the maximum PP is inferred as being the ML ancestral state. Site- and time-homogeneous models (LG and LG+F_opt_), site-heterogeneous (EX2, EX3, EHO, UL2, UL3, EX_EHO [Le, Lartillot, et al. 2008], C10–C60 [Le, Gascuel, et al. 2008], and time-heterogeneous models COaLA with 1 or 2 parameters per branch [Groussin et al. 2013] were run with bppML to retain the best-fitting model in terms of AIC [Akaike 1973] and BIC [Schwarz 1978] values). Ancestral gaps inferred by Prank were then incorporated in the final ancestral sequences.

Several models available in PhyloBayes 3.3 were used to compute the ancestral sequences: LG, GTR, CAT, and CAT+GTR. As the latter model generally provides the best fit to the data, its estimation provided us the ancestral sequences to be synthesized. MCMC chains were run for 1,000,000 cycles, saving a sample each 10 cycles and discarding the first 1,000 sample as burnin. Two independent chains were checked for convergence using the tracecomp program provided in PhyloBayes 3.3. Convergence is estimated to be reached when the difference of posterior log likelihood across MCMC chains is less than three times the estimated standard error. The ancestral sequences were computed from the posterior distributions using the ancestral program of the PhyloBayes 3.3 suite. We used the midpoint rooting option (-midpointrooting) to provide an estimate at the root node. For each site and ancestor, the considered ancestral state has the maximal PP. Ancestral sequences obtained from two independent MCMC chains were compared and verified to be identical. As final step of the ancestral sequence inference, ancestral gaps inferred by prank are substituted to the inferred ancestral states, yielding the ancestral amino acid sequences to be synthesized.

### Gene Synthesis, Protein Expression, and Purification

The complete gene synthesis of the various ancestral sequences was done by GeneCust (www.genecust.com). The resulting genes were cloned in pET 21a between the Nde1 and BamH1 sites. The resulting constructs were transformed in *Escherichia coli* BL21 DE3 pLysS strain. Cultivation in LB medium containing ampicillin, overexpression, refolding and purification were done accordingly to protocol used for *H. mari* MalDH (Irimia et al. 2003).

### Analytical Ultracentrifugation

Sedimentation velocity experiments were performed using a Beckman XL-I analytical ultracentrifuge and an ANTI 50 rotor (Beckman Coulter). In order to analyze the change in oligomeric state as a function of salt concentration, the experiments were carried out at 20 °C for all MalDH at 0.1 mg/ml in 50 mM Tris pH 7.0, supplemented with the appropriate salt concentration. A volume of 400 µl was loaded into double-channel centerpiece of 1.2 cm optical path length and centrifuged at 130,000 × g (42,000 rpm). Scans were recorded at 280 nm. The partial specific volume of the polypeptide chain for all MalDHs was  = 0.73 ml/g as calculated with the Sednterp software (free available at http://www.jphilo.mailway.com/). The solvent density, ***ρ***and the solvent viscosity ***η***at 20 °C for each tested conditions were calculated with the same software. Sedimentation profiles were analyzed by the size-distribution analysis of Sedfit (free available at http://www.analyticalultracentrifugation.com). In Sedfit, finite element solutions of the Lamm equation for a large number of discrete, independent species, for which a relationship between mass, sedimentation and diffusion coefficients, ***s*** and ***D***, is assumed, are combined with a maximum entropy regularization to represent a continuous size-distribution (Schuck 2000). We used 200 generated sets of data on a grid of 300 radial points, calculated using fitted frictional ratio for sedimentation coefficients comprised between 1 and 10 S. For the regularization procedure, a confidence level of 0.68 was used.

### Conformational Stability

Residual activity measurements using the standard assay (done in triplicates) and circular dichroism (CD) were used to determine conformational stability as in previous study (Ebel et al. 1999; Madern, Ebel, Mevarech, et al. 2000). For the determination of salt-dependent conformational stability, the proteins of interest were incubated at 20 °C for 24 h at 0.1 mg/ml in various salt concentrations buffered with 50 mM Tris–HCl pH7 prior to performing CD and activity assays. The data were expressed as % of the values recorded before incubation. The salt concentration at which 50% of the protein is still folded and active after incubation (*M^f^*_½_) was graphically determined using the transitions curves. The confidence limits are ±0.1 M in KCl, CsCl, and KF. For the pH-dependent conformational stability, the protein were incubated at 2 M KCl with various buffers at 100 mM, that is, sodium citrate for pH5, MES for pH6, Tris–HCl for pH7 and pH8 and pH9, glycine for pH10, borate-NaOH for pH11, Na_2_HPO_4_ for pH12 and NAOH for pH13. Far-UV CD measurements were carried out on a JASCO J-810 thermostated spectropolarimeter. Far-UV spectra were recorded in 0.1-cm path length quartz cells. The spectra shown in this work represent the average of three accumulated consecutive scans.

### Standard Enzymatic Assay

The activity was determined at 25 °C, by following the oxidation of NADH over 30 s by measuring the decrease in absorbance at 340 nm (Beckman DU 7500 Spectrophotometer) in 3.8 M KCl, 50 mM Tris–HCl buffered at pH7 supplemented with 1.0 mM OAA and 0.2 mM NADH. To test the substrate inhibitory effect, measurements were done at various concentration of OAA.

## Supplementary Material

Supplementary data are available at *Molecular Biology and Evolution* online.

## Supplementary Material

msab146_Supplementary_Data
