# Supplementary material for: Resurrection of Ancestral Malate Dehydrogenases Reveals the Evolutionary History of Halobacterial Proteins: Deciphering Gene Trajectories and Changes in Biochemical Properties
Source: Mol Biol Evol. 2021 May 11;38(9):3754–74. doi: 10.1093/molbev/msab146 (PMC8382911; doi:10.1093/molbev/msab146)
Supplement: msab146_Supplementary_Data [file msab146_supplementary_data.zip › Answer to reviewers 14 03 2020.pdf]

Reply to Reviewer: 1.

R1.1. Blanquart et al. report an ancestral sequence reconstruction study into the evolution of halobacterial proteins. The authors perform a diversity of biophysical determinations on modern and ancestral malate dehydrogenases. However, no clear message of general interest seems to emerge from these studies or, at least, the authors fail to make the message clear to the reader.

Reply. The main findings that we estimate to be of wide interest are now better highlighted in the Introduction and Conclusion. First the use of the ALE method yield a complex evolutionary history by taking into account HGT in ASR, suggesting that current ASR studies would be biased if complex processes are not accurately accounted for. Second, our results challenge the current model of H. marismortui MalDH as a typical enzyme for understanding halophilic adaptation, as well as the current view on Halobacteria evolution. Finally the lost inhibition phenotype concomitant to the secondary adaptation to halkaliphily may document a more general evolutionary tradeoff.

We would also mention that the opinion of the reviewer R1 concerning the general interest of our work is not shared by the three other reviewers.

R1. 2. Such evolutionary narrative is completely missing from the submitted manuscript (or, again, the authors fail to make it clear to the reader).

Reply. As mentioned above, although focusing on a precise study case, our study also provides a wider picture on the Halobacteria evolution. We tried to make these points clearer in the ms.

R1. 3. In fact, the manuscript is plagued with over-detailed and ambiguous discussions on very specific issues, whose relevance is difficult to appreciate.

Reply. To our opinion, the understanding of complex phenomenons requires in depth investigation; consequently the analysis of data cannot be superficial. To this respect, our work focuses on how an enzyme adapted to extremophilic lifestyle by analyzing ancestral phenotypes at the molecular level. Although the question of “where” or “when” organisms evolved is fundamental, we believe that the question of “how” it happens is also of a wide interest. Some parts of the text has been changed in order to better convince the reader that our molecular level analyses yield insight into the Halobacteria evolution.

R1. 4. On the other hand, issues of general interest, such as how halophilic features emerged from non-halophilic proteins, do not seem to be addressed.

Reply. We have also taken into account this remark. We have more clearly indicated that there is no correlation between the KCl concentration dependent stability of present day MalDHs with respect to the NaCl concentration of the environment. A fact that prevents the construction of a salinity sensor to infer the paleoenvironment in which a primitive halobacterial organism would have emerged. This is presented as a specific paragraph in the Discussion in the new version of the manuscript. Moreover, during the evaluation process of our manuscript, a work by Martijn et al. has been published that relates, in Archaea, the transition from methanogen to halophiles. We have clearly mentioned this work, which is based on comparative genomics, for readers interested in the evolution from non halophilic to halophilic Archaea.

R1. 4. Also, some of the interpretations provided by the authors are questionable, such as when they state (page 32) that "the accumulation of destabilizing mutations...may render the enzyme prone to evolve new properties". As it is well known, it is high stability (and accumulation of stabilizing mutations) which contributes to evolvability, as it is actually proposed in the paper of Frances Arnold and coworkers (Bloom et al., 2006) that the authors reference on page 32 of their manuscript.

Reply. We have better discussed our data. In particular, we added more references showing that destabilizing mutations cannot be ignored as also playing a role in the evolution of new properties. A phenomenon that is due to the buffering effect of chaperones, as it is shown by the work of Geller et al., 2018 added in reference. This is reported in the Conclusion, second paragraph.

Reply to Reviewer: 2.

The manuscript by Blanquart and colleagues describes their attempt to dissect the evolutionary history of malate dehydrogenase in a halobacterial lineage via ancestral sequence reconstruction. The authors utilize a variety of molecular evolutionary/bioinformatic tools to dissect this gene family do to a semi-complex history of duplications, loses and horizontal transfers. Although I do not have expertise in halophilic protein evolution, the results seem straightforward to interpretation in terms of solubility, stability and enzymatic activity. I do, however, have some concerns:

R2.1. The reader does not have a sense for the amount of sequence evolution between ancestors. For example, there is a major phenotypic shift between Anc88 and Anc89 (Fig. 6) but we have no sense of the sequence divergence between these two. I can see from the supplemental material that the sequences have identical lengths but how many aa differences do they have? And the rest of the ancestors?

Reply. We thank the reviewer for this suggestion. In order to count the number of amino acid replacements between each sequences, the alignments between the ancestral and modern enzymes, for the three lineages we investigated, were added in Supplementary Material part. They allow seeing the location at which the replacements occurred. In Fig 3, we added a small inset that helps the reader to see the direction of evolution from the last common ancestral enzyme. The figure legend is now more explicit. For the specific Fig 6, we have added the numbers of AA replacements, as requested. We have also added the numbers of AA replacements for all ancestors in the Fig 9, which summarizes the trajectory.

R2.2. The phenotype of Anc88 is not provided in Fig S5A. Was this oversight or did the protein not function in this condition?

Reply. We thank the reviewer for his/her detailed inspection of the ms. The fig S5A suffered a wrong annotation, it is now fixed.

R2.3. Why does the legend of S4 say ‘stars indicate aa differences’?

Reply. It was a typo error, which is fixed.

R2.4. There is no explanation of the tree scale in S3.

Reply. The explanation is now included in the legend.

R2.5. I appreciate that the ASR analyses used multiple MCMC runs to determine convergence. However, I do not recall any reference to convergence for the phylogenomic analysis.

Reply. More explanations are now included in Materials and Methods, in a first new paragraph "Main phylogenetic reconstruction steps" summarizing the reconstruction steps, and also in the relevant M&M sections.

Can we be confident of the results if there was no test for convergence?

Reply. Details on MCMC convergence assessment for each bayesian experiment are now added to the text. No convergence difficultyconcerns the ASR step: there are few and short sequences to analyze, the tree topology is fixed. For this step we assessed that the ancestral sequences reconstructed with two independent MCMC chains are identical, which is now mentioned in the text. MCMC convergence of the phylogenomics experiment (yielding the species tree given a large set of sites) is more difficult. In this case the considered tree is therefore obtained using ML, and bayesian models were used only as controls. Convergence criteria were already mentioned in the text. To make the reader confident with the protocol, we provide now a brief summary of the phylogenetic reconstruction main steps at the Methods beginning.

R2.6. The authors have done a reasonable job of hypothesizing about the HGT of MalDH to H. mari., and this may or may not reflect biological reality. As such, I think the authors should briefly discuss the possibility that LBA is driving the gene-tree results despite the authors best attempt to account for its potentially misleading topological effects.
Reply. Branch length and posterior probabilities indicates that LBA cannot be considered as a phenomenon responsible for the observed tree topology. We also recall that, the biochemical properties observed gives strong experimental support to this tree topology. These points are more clearly indicated in Results, second paragraph.

R2.7. There are numerous grammatical errors throughout the manuscript. I appreciate most of the authors are not native English speakers so it is important to have the manuscript proofread because I was sometimes confused by the wording.

Reply. In order to fix the errors, the manuscript was corrected by a native english speaker.

Reply to Reviewer: 3.

This paper uses ancestral protein sequence resurrection and biochemical analysis to probe the molecular evolutionary history of malate dehydrogenase (MalDH) in extreme halophilic Archaea. The authors resurrect nine ancestors and compare their biochemical properties with five extant Halobacterial MalDHs. The authors carried out extensive biochemical characterizations on these ancestral and modern proteins, including stability in different salts and salt concentrations, oligomeric states, and enzymatic properties. While the text of the manuscript can benefit from some further editing and streamlining, this paper presents a lot of valuable experimental data, generating numerous interesting hypotheses which can be further tested and studied in future work. I have a few minor comments described below.

We thank the referee.

R.3.1. The paper presents a lot of biochemical data on ancestral versus modern proteins, but what is not clear is how much of these biochemical data can be rationalized in terms of sequence changes within the context of the 3D structure. It appears that sequence/structure analysis is treated in a separate companion paper ("Part 2"), and the Discussion of the current paper ("Part 1") provides a good summary of a few cases where the biochemical data can indeed be rationalized in terms of a small number of changes in protein sequence within the context of 3D structure. But can all of the biochemical data be rationalized in such a clean way, by a small number of sequence changes within the 3D structure? Are there any biochemical observations that are particularly puzzling, from the perspective of sequence/structure analysis? The paper can benefit from more discussion on this.
Reply. The objective to rationalize the data within the context of the 3D structure would be achieved providing the companion Ms-part 2 had been accepted. Unfortunately, this is not the case. Consequently, we suppressed the few details (and references to the unpublished yet work of Girard et al) mentioned with respect to the new crystal structure of Haloferax volcanii solved with its solvation shell. Instead of using this new unpublished yet structure, we now use the one of Haloarcula marismortui, solved in previous work but with only few ions. This do not change our findings and analysis, but means we now employ conditional usage of verbs and provide less detailed analysis with respect to the ions. See for example the last paragraph of "The halo-alkaliphilic phenotype of MalDH is a secondary evolutionary trait" part.

We wish to submit the second manuscript as soon as possible to reinterpret our data in the light of the Haloferax volcanii MalDH structure described with its solvation shell and binding ions.

R3.2. The Abstract reads: "Our results demonstrate that subtle complementary changes in Halobacterial MalDH sequences resulted in the independent divergence of solubility, stability and enzymatic properties of the three lineages of Halobacteria". What does "complementary changes" mean? This term is used only once in the Abstract, and never again in the body of the manuscript.

Reply. We agree that it was an inappropriate term. We have slightly reorganized the Abstract in order to present our data without any ambiguities.

R3.3. Approximately 20% of the biochemical data are missing (marked as "ND" or "Not Determined" in Figure 9). What is the mechanism of missing data? Is this because the biochemical experiment was not performed in the first place? Or is it because the biochemical experiment was performed but failed? And if so, for what reason(s)? The mechanism of missing data is important for subsequent data analysis, and I suggest that the authors include a detailed description of the mechanism of missing data, and if there is more than one mechanism of missing data, list them explicitly for different "ND" entries.

Reply. This is a commendable request, but, on one hand we cannot add so detailed information for experiments that were not successful, whereas on the other hand the rest of referees said that there is almost too numerous data ! Thus, we now mention ND1 for folding problems preventing a characterization, and ND2 when experiments failed or were not done.

R3.4.The authors mentioned "long-range effect" several times, but it is not clear what it means. The authors should clearly define what "long-range effect" means. In cases where sequence changes are not located at the corresponding functional sites, are the sequence changes close to or far away from the functional sites? How long-range is considered long-range enough? And what would be the possible mechanisms for such "long-range effect"?

Reply. The term **"**long range effect" is of trivial usage in the field of structural biology. It refers to the dynamical properties of proteins that cannot be considered as static molecules and for which it exists a huge amount of data. These dynamical properties implies pico to milli second structural reorganizations that influence either the stability, the activity or both at the same time. We hope that the new small paragraph added at the end of the part " Molecular evolution in Halobacteria, a case of study" subsection "*Conformational stability,* provides a satisfactory explanation. We also added two references that would help readers interested by such a phenomenon.

Reply to Reviewer: 4.

"Resurrection of Ancestral Malate Dehydrogenases Reveals the Evolutionary History of Halobacterial Proteins. Part 1." by Samuel Blanquart, Mathieu groussin, Aline Le Roy, Gergely J Szollosi, Eric Girard, Bruno Franzetti, Manolo Gouy and Dominique Madern, look at the evolutionary history of an enzyme, malate dehydrogenases in Halobacteria, which are a group of related archea that are adapted to high salt environments. They built species trees and gene trees for Halobacterial malate dehydrogenases and reconciled them. Using these reconciled trees, they did ancestral sequence reconstruction and identified ancestral sequences at the nodes. What is compelling about their study, was that they went on to actually build these ancestral enzymes and compared their 3D structure and behavior with that of the modern enzymes in different salt solutions of varying concentrations. They were able to compare the amino acid composition of the homologous enzymes that affects their interaction with their solvent environment. Overall, my impression of this work is that it is an important exploration of the connection between genotype and phenotype through an evolutionary and phylogenetic lens.

R4.1. Addressing a sentence or two on the effect of regulation on these enzymes on their function in the introduction would provide a more complete picture of the links between genes and their phenotype.

Reply. As requested, we have added some appropriate sentences at the end of the second paragraph in the introduction

R4.2. Did you only make the ML ancestral sequences or did you also make proteins with alternative amino acid sequences based on other methods?

Reply. The synthesized ancestral MalDH sequences were computed using a bayesian model, not ML. Controls were realized using ML methods, showing very few differences with the bayesian estimated. To make our phylogenetic reconstructions clearer to the reader, we add a brief summary of the main reconstructions steps at the beginning of the Methods. Due to the synthesis and characterization cost, control ML ancestral sequences were not analyzed.

R4.3. When looking at the DE/RK ratios, were you looking at specifically surface amino acids, or total amino acids. From what I can tell, the ratio was involving all interactions, but your conclusions from the results were all about surface enrichment. Were you able to differentiate between surface amino acids for your analysis?

Reply. We added a specific paragraph (6 lines) at the beginning of the part "Evolution of surface residues and protein solubility". For readers interested by the calculation, the reference of Coquelle et al., is given.

R4.4. In Figure 2, the image of the 3D structures are covering the bars, so it's difficult to make out where all the bars actually stop.

Reply. We have displaced the 3D images allowing to see clearly the whole bars.

R4.5. I think it's necessary to provide a little bit more reasoning for why those specific salts were chosen.

Reply. As requested we have changed the paragraph at the beginning of the part "Salt concentration dependence of conformational stability in modern and ancestral MalDHs." It explains more clearly that playing with different salts is a way to probe the relative effect of ions. In solution, a salt does not longer exist. It dissociates and consequently a protein containing solution becomes a complex mixture made of protein, water, hydrated cation and hydrated anions, which are in equilibrium. Thanks to this, one can analyze fundamental properties of a given anion or cation by choosing a set a salt. Owing to extensive characterization of H. marismortui MalDH, we know which salts are immediately informative with respect to the stabilization process. In such a framework, we mention clearly that these studies requested the use of non-physiological salt.

R4.6. dN/dS tests to look for evidence of selection would help support your findings connecting selection to the phenotypic and genotypic differences observed.

Reply. A sentence that refers to the use of dN/dS tests was added at the end of the second paragraph, in "Distinct evolutionary trajectories on the way to modern halobacterial MalDHs". It mentioned that inferring dN/dS ratio would require wider taxonomic sampling and dna sequences which, we believe, is out of the scope of our study.

R4.7. When building gene families from a blast search for homologous genes, you chose to include all that have more than 30% coverage. This number seems quite small to me, and 80% similarity seems like it may be a bit too high for this time scale? Please defend the use of these cut off values.

Reply. We thank the referee for her/his detailed reading: the numbers that we mentioned previously were erroneous due to an inversion. It is now fixed: "30% similarity and more than 80% coverage" in the section reconstruction of gene families "Materials and Methods"

R4.8. . Please clarify if indels were considered a 21st character state with neighboring indels treated as independent, and if that assumption affects your reconstruction.

Reply. None of the used phylogenetic models applied in our work, except Prank, use gap as an independent 21st state. Prank yields the final MalDH alignment accounting for insertion and deletion events (gaps as states). No indels are estimated within halobacterial MalDHs, which all have the same length, and insertions are estimated in the outgroup MalDHs. Our last step reconstructs ancestral MalDH sequences using the CAT+GTR bayesian model, which does not account for gaps as states and estimates states at sites corresponding to the outgoup insertions. These states should not be considered in the ancestral halobacterial MalDH and are discarded according the the output Prank alignment. Consequently, there should have very limited effects of ancestral indels on our estimates. These points were already mentioned in the Methods and Supplementary Material sections. However to make all the phylogenetic reconstruction steps clearer to the reader, we had a brief summary of main steps at the beginning of the Method section. It highlight that Prank considers gaps as states.
