# Supplementary material for: Resurrection of Ancestral Malate Dehydrogenases Reveals the Evolutionary History of Halobacterial Proteins: Deciphering Gene Trajectories and Changes in Biochemical Properties
Source: Mol Biol Evol. 2021 May 11;38(9):3754–74. doi: 10.1093/molbev/msab146 (PMC8382911; doi:10.1093/molbev/msab146)
Supplement: msab146_Supplementary_Data [file msab146_supplementary_data.zip › Blanquart et al Supp. Mats MBE resubmited 14 03 2020.pdf]

Supplementary information

Resurrection of Ancestral Malate Dehydrogenases Reveals the Evolutionary History of Halobacterial Proteins : Deciphering Gene Trajectories and Changes in Biochemical Properties.

Samuel Blanquart[1], Mathieu Groussin[2, 3], Aline Le Roy[4], Gergely J Szöllosi[2, 5], Eric Girard[4], Bruno Franzetti[4], Manolo Gouy*[2] and Dominique Madern*[4]

1. Univ Rennes, Inria, CNRS, IRISA, Rennes F-35000, France

2. Université Lyon 1, CNRS, UMR5558, Laboratoire de Biométrie et Biologie Évolutive, 43 bd du 11 novembre 1918, F-69622 Villeurbanne. France.

3. Center for Microbiome Informatics and Therapeutics, Massachusetts Institute of Technology, Cambridge, Massachusetts 02139. USA

4. Univ Grenoble Alpes, CNRS, CEA, IBS, F-38000 Grenoble. France

5. MTA-ELTE "Lendulet" Evolutionary Genomics Research Group, H-1117 Budapest, Hungary.

***Corresponding authors:** E-mail: manolo.gouy@univ-lyon1.fr, dominique.madern@ibs.fr

Selection of species

Ingroup

We used a set of 67 Halobacteria genomes available in GenBank. The malate dehydrogenase gene family was then reconstructed with Silix (Miele *et al.* 2011) (see below) and species lacking MalDH were removed from the dataset. Besides, several genomes contain nearly identical MalDH sequences. We randomly selected a MalDH sequence among these species to avoid taxonomic redundancy and controlled for the influence of the random choice on the reconstruction of the species tree. Finally, three species (*Halobacterium salinarum R1*, *Natronomonas pharaonis* DSM 2160 and *Halalkalicoccus jeotgali* B3 5435) were discarded from the dataset due to difficulties to confidently decipher their phylogenetic position in the species tree. 28 halobacterial species remained in the final dataset. Only two species (*Haloterrigena thermotolerans* and *Natronolimnobius innermongolicus*) possess a duplicate gene of MalDH.

Outgroup

A taxonomically-rich outgroup was considered during the reconstruction of the species tree and MalDH trees. We considered the archaeal species tree reconstructed in (Brochier-Armanet *et al.* 2011) to select 21 euryarchaeal species outside of Halobacteria. We retained the sequenced genomes of the two Nanohaloarchaea species (*Candidatus Nanosalinarum* sp. J07AB56 and *Candidatus*

Nanosalina sp. J07AB43). Recent phylogenetic analyses showed that Nanohaloarchaea and Halobacteria derive from two distinct methanogen ancestors (Aouad *et al.* 2018).

Phylum	Species Name
Haloarchaea	Halorhabdus utahensis DSM 12940
	Halosimplex carlsbadense
	Haloarcula marismortui ATCC 43049
	Halomicrobium mukohataei DSM 12286
	Halorubrum hochsteniumATCC 700873
	Halorubrum arcis
	Halorubrum californiensis
	Halorubrum aidingense
	Halorubrum lacusprofundi ATCC 49239
	Haloferax volcanii DS2
	Halogeometricum borinquense DSM 11551
	Haloquadratum walsbyi DSM 16790
	Halovivax asiaticus
	Natronococcus amylolyticus DSM 10524
	Natronococcus jeotgali
	Natrialba aegyptia DSM 13077
	Natrialba magadii ATCC 43099
	Haloterrigena limicola JCM 13563
	Haloterrigena thermotolerans
	Natrinema altunense
	Natronorubrum tibetense
	Natronorubrum bangense
	Natronorubrum sulfidifaciens JCM 14089
	Haloterrigena turkmenica DSM 5511
	Natronolimnobius innermongolicus
Nanohaloarchaea	*Candidatus NanoSali* Sp

	Candidatus NanoSalinarium Sp
Methanocellales	*Methanocella paludicola* SANAE
	Uncultured Methanogenic Archaeon RCI
Methanosarcinales	*Methanosaeta thermophila* PT
	Methanosaeta concilii GP6
	Methanosarcina barkeri str. Fusaro
	Methanosarcina mazei Go1
	Methanosarcina acetivorans C2A
	Methanococcoides burtonii DSM 6242
	Methanohalophilus mahii DSM 5219
	Methanohalobium evestigatum Z 7303
	Methanosalsum zhilinae DSM 4017
Methanomicrobiales	*Methanocorpusculum labreanum* Z
	Methanoplanus petrolearius DSM 11571
	Methanoculleus marisnigri JR1
	Methanospirillum hungatei JF-1
	Candidatus Methanoregula boonei 6A8
	Methanosphaerula palustris E1 9c
Archaeoglobales	*Ferroglobus placidus* DSM 10642
	Archaeoglobus fulgidus DSM 4304
	Archaeoglobus profundus DSM 5631
	Archaeoglobus veneficus SNP6
Thermoplasmatales	*Thermoplasma volcanium* GSS1
	Thermoplasma acidophilum DSM 1728
	Picrophilus torridus DSM 9790

Table 1:Species considered in the protein concatenate.

Species tree reconstruction

The species tree (shown as a pipe in Fig.1) was obtained by using PhyML 3.0 (Guindon *et al.* 2010)

from a large phylogenomic data matrix consisting of 50135 amino acid sites and 51 archaeal species and applying the empirical site and time homogeneous model LG (Le and Gascuel 2008, see Methods). The obtained species tree displays an identical topology (with stronger support for the main nodes of divergence) than the one obtained by Becker *et al* 2014, with three main lineages denoted by: clade A (including *Halomicrobium mukohataei* and *Haloarcula marismortui*), clade B (including *Haloferax volcanii*) and clade C (including *Natrialba magadii*, shown in the reconciled tree (Fig.1). Although deepest nodes in the species tree are weakly supported (bootstrap support for B+C: BP=55%), all other nodes are strongly supported with BP=100% (Fig.S1). Most noticeably, another resolution of the grouping of clade A, B and C was obtained by Brochier-Armanet *et al.* (2011), in which Halobacteria clades A and C group together with strong bootstrap support. In order to decipher among such incongruities, we applied three distinct strategies shown to decrease reconstruction bias. First, we applied the slow-fast method (Brinkmann *et al.* 2005, Philippe *et al.* 2005), consisting in the gradual suppression of fastest evolving sites from the alignment in order to avoid long-branch attraction (LBA) artifacts (see Methods). Once 20% and more of the fastest evolving sites are removed, the grouping of clade B and C appears to be strongly supported (LG model, BP>96% (FigS1).

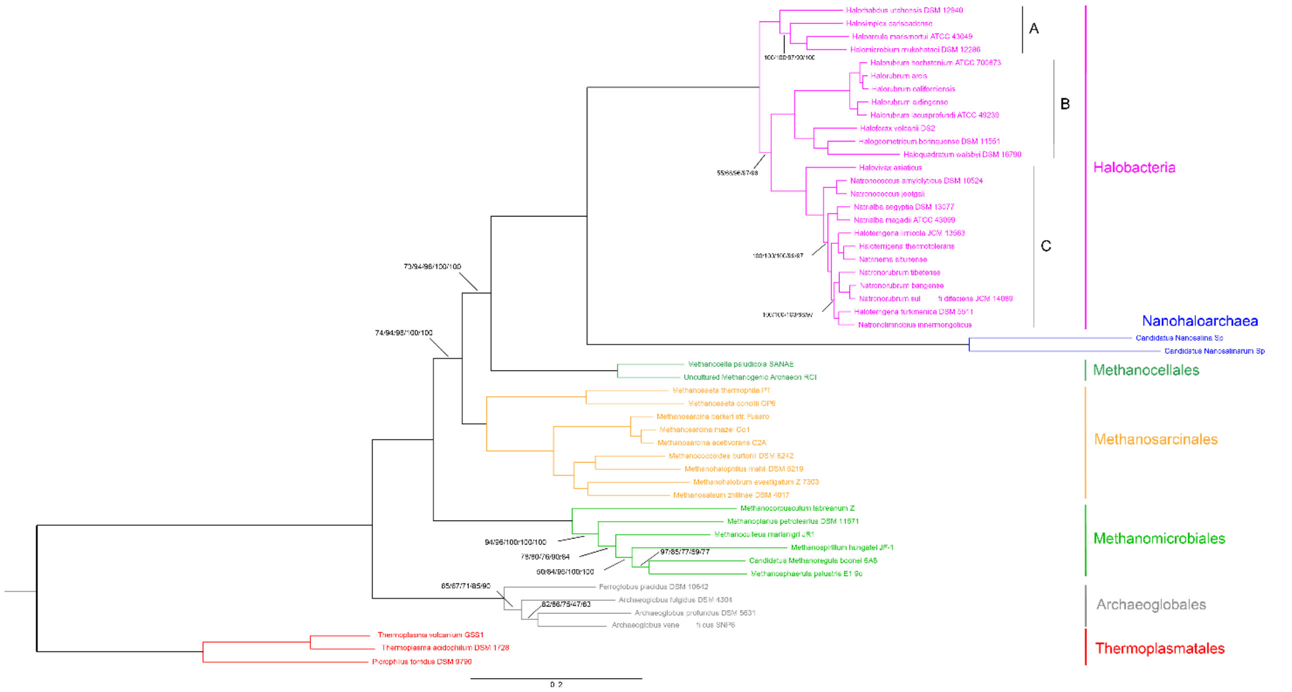

FIG.S1. Maximum Likelihood phylogenetic tree of the 51-archaeal species. The tree was reconstructed with a ML approach using PhyML and the LG+Γ(4) model. The alignment contains 51 archaeal species and 50,135 sites. The different phyla are color-coded. The support for the tree was evaluated with a bootstrap approach, using 100 replicates. Branches with no support information have 100% bootstrap support. When bootstrap values are indicated, they correspond to the result obtained with an elimination of 0%/10%/20%/30%/40% of the fast-evolving sites originally present in the

dataset. In each case, the ML topology was inferred to be same. The species was rooted following Brochier-Armanet *et al*. (2011).

Moreover, we used the site-heterogeneous CAT model (Lartillot and Philippe 2004), which was proved to alleviate LBA artifacts. Although we were not able to obtained Markov Chain Monte Carlo (MCMC) convergences with the largest datasets, the replicate with 50% of removed fastest evolving sites also provided strong support for the grouping of clades B and C (posterior probability PP=1). Moreover the obtained topology within Halobacteria is identical to that obtained with LG (Fig.S1) and strongly supported (all PP=1).

Finally, we tested the relationships at the root of Halobacteria to potential compositional biases by using the time-heterogeneous model CoaLA (Groussin *et al.* 2013). Anew, as 20% or more of the fastest evolving site are removed, AU tests for the root position strongly support a grouping of clade B and C (AU>0.96, see Table S2). The results led us to confidently root the Halobacteria tree on the branch leading to clade A, and to group together clades B and C (Ancestral node Anc78, see Fig.1.)

	AU values		
% of sites eliminated	Root A	Root B	Root C
0 %	0.728	0.295	0.005
10 %	0.809	0.198	0.001
20 %	0.961	0.040	2e-04
30 %	0.982	0.018	3e-04
40 %	0.985	0.015	1e-04
50 %	0.996	0.004	2e-04

Table S2: Topology comparison for the inference of the root position of Halobacteria with COaLA. Root X correspond to a root where group X emerges first in Halobacteria (Fig.1). Values in the table correspond to the results of AU tests of topology comparisons.

MalDH tree reconstruction

The MalDH alone gene tree was computed using the Bayesian CAT+GTR model (Lartillot *et al.* 2009) from the amino acid alignment. The model implements a mixture of free amino acid profiles distributed across sites, together with free instantaneous replacement rates between amino acid pairs. This model was preferred over available empirical models because halobacterial proteins display a strong acidic amino acid enriched composition, which presumably is due to a peculiar pattern of amino acid replacements. As expected, considering single gene analysis and underlying stochastic

errors, the obtained CAT+GTR MalDH tree globally appeared to be weakly supported (Fig.S2). Moreover, it was highly incongruent with the previously obtained species tree: a Robinson-Foulds distance of 28 indicated that two thirds of its bipartitions were not observed in the species tree. Furthermore, *Natronolimnobius innermonglolicus* and *Haloterrigena thermotolerans* each possess two copies of MalDH and one copy set clustered outside the monophyly of all other MalDH with weak support (PP=0.6, Fig.S2). Additionally, no MalDH was found in the genome of *Halorhabdus utahensis*. These observations indicate that halobacterial MalDHs did not follow an orthologous evolution and encountered events such as gene duplication, transfer and loss (DTL) occurring during their evolution.

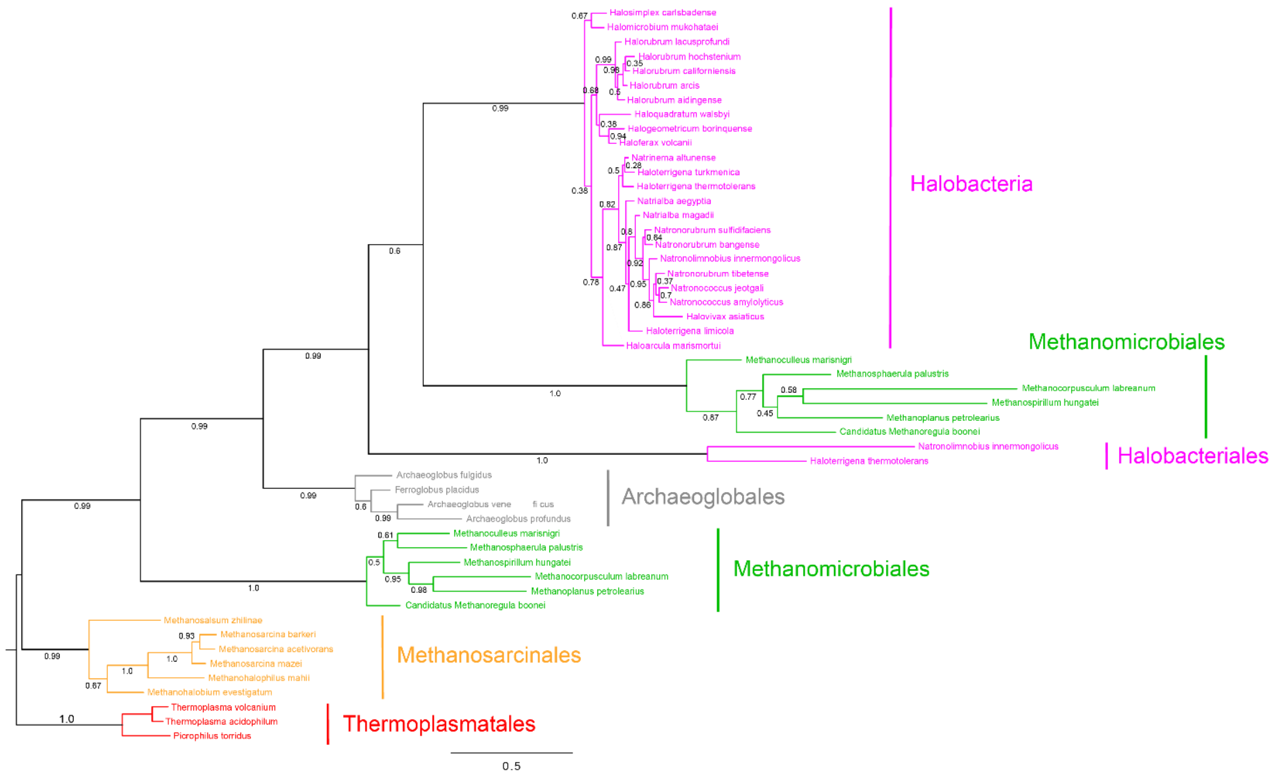

Fig.S2. Sequence-only MalDH phylogeny. The tree was inferred with Phylobayes 3.3 and the CAT+GTR model. Supports on branches are posterior probabilities. Scale bar indicates the expected number of amino acid replacement per site.

Therefore, we used the ALE method (Szöllosi *et al.* 2013b), implementing the exODT model (Szöllosi *et al.* 2013a) to construct a reconciled MalDH phylogeny accounting for probabilistic DTL events, given a guide species tree (see Methods). The obtained MalDH joint-tree (Fig.1) is much closer to the species tree (Robinson-Foulds of 16), suggesting that most of the discrepancies between the CAT+GTR MalDH tree and the species tree indeed arose due to a lack of phylogenetic signal.

Most noticeably, the MalDH joint-tree clusters the highly diverged MalDH copies of

Natronolimnobius innermonglolicus and *Haloterrigena thermotolerans* at the base of all over halobacterial MalDH, indicating horizontal gene transfer (HGT) from an old and possibly extinct Halobacteria lineage. Importantly, this estimated oldest ancestor of halobacterial MalDH was not considered for the following ASR. Within the remaining tree, ALE inferred a single duplication, 14 losses and 3 HGTs. One HGT is estimated to have occurred from a lineage related to the ancestors of clade C toward *Haloarcua marismortui* in clade A (PP=0.99, Fig.S3). All over DTL events are estimated to have occurred within clade C. Thus, except for the *Haloarcula marismortui* MalDH, all MalDH in clade A and B are estimated to have followed an orthologous evolution. A control with the parsimony based methods Mowgli (Nguyen *et al.* 2013) inferred 8 losses and 10 LGT events, which confirmed that halobacterial MalDH indeed experienced a complex evolution, which should not be assimilated to the Halobacteria species tree.

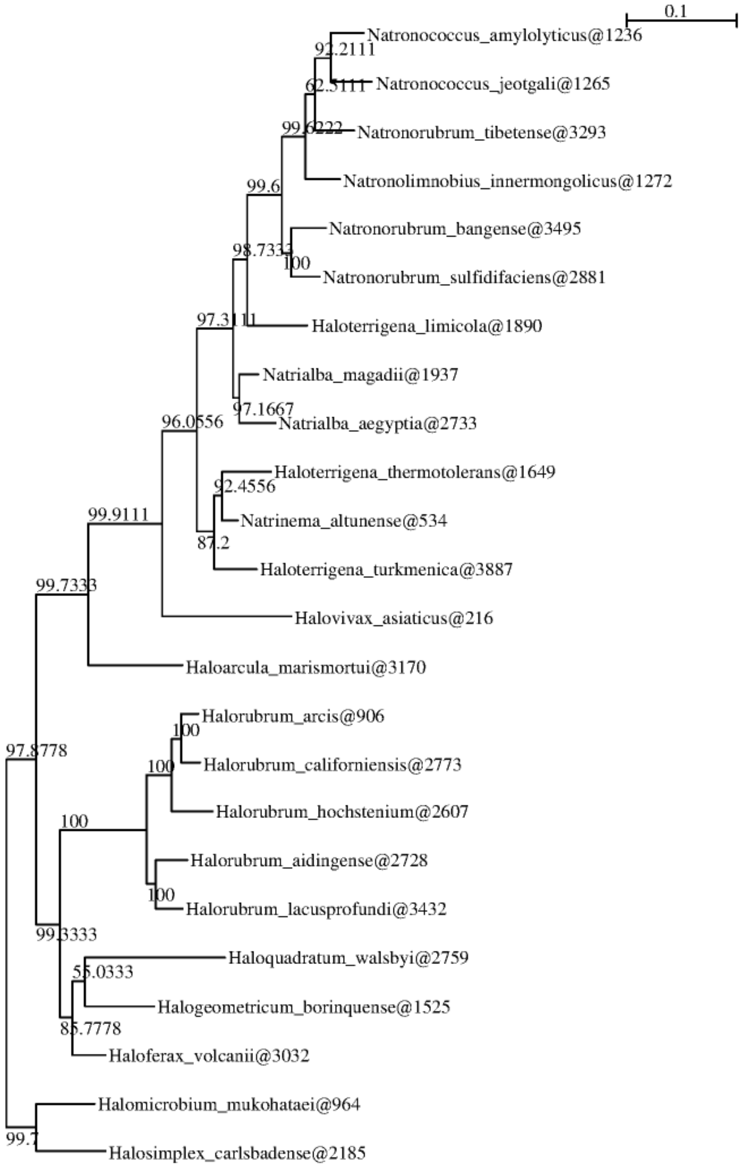

FIG.S3. Joint tree of MalDH. The phylogenetic tree maximizing the joint likelihood of sequence and DTL events information was calculated with ALE. The scale bar indicates the expected number of replacement per site. This tree was used to reconstruct ancestral sequences of extant MalDHs (dashed lined tree in Fig.1).

Ancestral MalDH reconstruction

A final alignment was computed using PRANK (Löytynoja and Goldman 2008), considering the 51 MalDH and the MalDH joint-tree as guides. Ancestral MalDH sequences were computed using the CAT+GTR model (Lartillot *et al.* 2009) and ancestral indels were removed (see Methods). In all the 9 considered MalDH ancestors, most of the ancestral sites were inferred with strong support (PP>0.9), consisting in 90% of the sites for ancestor 89 (Anc89 MalDH) up to 96% of the sites in Anc84 MalDH,

and less than 5% of the ancestral sites were weakly supported (PP<0.8). Control experiments were performed using maximum likelihood reconstruction methods. Among 15 different tested models (see Methods), the site-heterogeneous model UL3 obtained the best fit to the data (AIC=29933.2, Table S3).

Process	Model	ln(Likelihood)	Number of Parameters	AIC	BIC
Site- and Time-homogeneous	LG	-15129.2	1	30260.4	30270.3
	LG+F_{opt}	-15075.4	20	30190.8	30269.81
Site-heterogeneous	UL2	-15011	2	30026	30033.9
	UL3	-14963.6	3	29933.2	29945.05
	EX2	-14999.3	2	30002.6	30010.5
	EX3	-14970.1	3	29946.2	29958.05
	EHO	-15022.6	3	30051.2	30063.05
	C10	-15433.6	10	30887.2	30926.71
	C20	-15357.6	20	30755.2	30834.21
	C30	-15307.1	30	30674.2	30792.72
	C40	-15243.6	40	30567.2	30725.23
	C50	-15252.4	50	30604.8	30802.33
	C60	-15229.3	60	30578.6	30815.64
Time-heterogeneous	LG+COaLA[1]	-15009.2	103	30224.4	30631.32
	LG+COaLA[2]	-14928.9	204	30265.8	31071.73

Table S3. Selection of the best-fitting model to reconstruct ML ancestral sequences of MalDH. LG+COaLA[*k*] indicates that the LG replacement matrix was considered when using the COaLA model, with *k* branch-specific parameters.

UL3 inferred with strong support (PP>0.9) from 93% up to 99% of the sites in Anc89 and Anc93 MalDHs, respectively. UL3 and CAT+GTR inferences were compared revealing very close reconstructions, with only 2 to 6 different ancestral sites inferred by the two models in Anc93 and Anc78 MalDHs, respectively (Fig.S4). All these differences occurred at sites weakly supported by CAT+GTR (PP<0.8). Biochemical characterization would highlight the true effects of such reconstruction discrepancies, but the comparison of different phylogenetic models and ASR protocols is beyond the scope of this work, and we only considered ancestral MalDH reconstructed by applying best available models and tools.

```
>Anc78 MalDH_CATGTR
MTKVSVVGAAGTVGAAAGYNLALRDIADELVFVDIPDQEDVTIGQAADTNHGVAYDSNTT
VRQGTYEDTAGSDVVVITAGIPRQPGQTRIDLAGDNAPIMDDIGSSLAEHNDDFVTITTS
NPVDLLNRHLYETGDRAREKVIGFGGRLDSARFRYVLSQRFDTPVQNVEATILGEHGDAQ
VPVFSKVRVDGTDPEFSADEKEAILGDLQESAMDVIERKGATEWGPATGVAHMVEAVLHD
TGEVLPGSVVLDGEFGHEDTAFGVPVKLGSNGVEEVVEWDLDDYEQDLMDDAAEKLSDQY
DKIA
```

>Anc78 MalDH_UL3
--------------------------V---------------------------------
--V---
--------------------------------------R----Q----------------
--I---
------------------------------D-----------------------------

>Anc80 MalDH_CATGTR
MTKVSVVGAAGTVGAAAGYNLALRDIADELVFVDIPDQEDVTIGQAADTNHGVAYDSNTT
VRQGGYEDTAGSDVVVITAGIPRQPGQTRIDLAGDNAPIMEDIGSSLAEHNDDFVTITTS
NPVDLLNRHLYETGDRAREKVIGFGGRLDSARFRYVLSQRFDAPVQNVEATILGEHGDAQ
VPVFSKVRVDGTDPEFSADEKEEILGDLQESAMDVIERKGATQWGPATGVAHMVEAILHD
TGEVLPGSVVLDGEFGHEDTAFGVPVKLGSNGVEEVVEWDLDDYEQDLMDDAAEKLSDQY
DKIA
>Anc80 MalDH_UL3
--------------------------V-------------T-------------------
--V---

>Anc81 MalDH_CATGTR
MTKVSVVGAAGTVGAAAGYNLALRDIVDELVFVDIPDQEDKTIGQAADTNHGVAYDSNTT
VRQGGYEDTAGSDVVVITAGIPRQPGQTRIDLAGDNAPIMEDIGSSLAEHNDDFVTITTS
NPVDLLNRHLYETGDRDRHKVIGFGGRLDSARFRYVLSQRFDAPVKNVEATILGEHGDAQ
VPVFSKVRVDGTDPEFSADEKEEILGDLQESAMDVIERKGATQWGPATGVAHMVEAVLHD
TGEVLPGSVVLDGEFGYEDTAFGVPVKLGSNGVEEVVEWDLDDYEQDLMDDAAEKLSDQY
DKIA
>Anc81 MalDH_UL3
--T-------------------
--V---

--I---

>Anc82 MalDH_CATGTR
MTKVSVVGAAGTVGAAAGYNLALRDIVDELVFVDIPDMEDKTIGQAADTNHGVAYDSNTT
VTQGGYEDTAGSDVVVITAGIPRQPGQTRIDLAGDNAPIMEDIGSSLAEHNDDFVSITTS
NPVDLLNRHLYETGDRDRHKVIGFGGRLDSARFRYVLSQRFDVPVKNVEATILGEHGDAQ
VPVFSKVRVDGTDPEFSADEKEEILGDLQESAMDVIERKGATQWGPATGVAHMVEAVLHD
TGEVLPGSVVLDGEFGYEDTAFGVPVKLGSNGIEEVVEWDLDDYEQDLMDDAAEKLSDQY
DKIA
>Anc82 MalDH_UL3
--V-----------------
--V---

--I---
--------L---R---

>Anc84 MalDH_CATGTR
MTKVSVIGAAGTVGAAAGYNLALRDVVDELVFVDIPDQRETTIGQAADTNHGVAYDSNTT
VRQGEYEDTAGSDVVVITAGIPRKEGQTRIDLAGDNAPIMEDIGSSLAEHNDDFVTVTTS
NPVDLLNRHLYEAGDRDRHKVIGFGGRLDSARFRYVLSQRFDAPVKNVEATILGEHGDAQ
APVFSKVRVDGRDPSFDADEKEEILEDLQESAMDVISRKGATQWGPATGVAHTVEAVLND
TGEVLPCSVVLDGEFGYEDTAFGVPAKLGSNGVEEVVEWDLDEYESDLLDEAAEKLSEQY
EKIA
>Anc84 MalDH_UL3

------A---

--I---
---------------------L------------------------E-------------

>Anc88 MalDH_CATGTR
MTKVSVVGAAGTVGAAAGYNIALRDIADELVFVDIPDQEDDTIGQAADTNHGVAYDSNTT
VRQGGYEDTAGSDVVVITAGIPRQPGQTRIDLAGDNAPIMEDIGSSLAEHNDDFVTITTS
NPVDLLNRHLYETGDRAREKVIGFGGRLDSARFRYVLSQRFDAPVQNVEATILGEHGDAQ
VPVFSKVRVDGTDPEFSADEKEELLGDLQESAMDVIERKGATQWGPATGVAHMVEAILHD
TGEVLPASVKLDGEFGHEDTAFGVPVKLGSNGVEEVVEWDLDDYEQDLMDDAAEKLSDQY
DKIA
>Anc88 MalDH_UL3

```
--------------------L-----V-------------T-------------------
--------------------------------------------------------V---
------------------------------------------------------------
------------------------------------------------------------
------------------------------------------------------------
----
>Anc89 MalDH_CATGTR
MTKVSVVGAAGTVGAAAGYNIALRDIADELVFVDIPDQEDDTIGQAADTNHGVAYDSNTT
VRQGGYEDTAGSDVVVITAGIPRQPGQTRIDLAGDNAPIMEDIGSSLAEHNDDFVTVTTS
NPVDLLNRHLYETGDRAREKVIGFGGRLDSARFRYVISQRFDAPVQNVEATILGEHGDAQ
VPVFSKVRVDGQDPEFDEDEKEELLGELQESAMNVIEKKGATQWGPATGVAHMVEAILRD
TGEVLPASVKLDGEFGHEDTAFGVPVKLGSNGVEEVVEWDLTDYEQDQLDEAAEKLSEQY
DKIA
>Anc89 MalDH_UL3
--------------------L-----V-------------T-------------------
------------------------------------------------------------
------------------------------------------------------------
------------------------------------------------------------
------------------------------D-----------------------------
----
>Anc90 MalDH_CATGTR
MTKVSVVGAAGTVGAAAGYNIALRDIADELVFVDIPDKEDDTIGQAADTNHGAAYDSNTT
IRQGGYEDTAGSDVVVITAGIPRQPGQTRIDLAGDNAPIMEDIGSSLAEHNDDFVTVTTS
NPVDLLNRHLYETGDRAREKVIGFGGRLDSARFRYVISQRFDAPVQNVEATILGEHGDAQ
VPVFSKVRVDGQDPEFDEDEKDELLEELQTSAMNVIEKKGATQWGPATGVGHMVEAILRD
TGEVLPASVKLEGEFGHEDTAFGVPVKLGSNGVEEVVEWDLTEYERNQLGEAAEKLSEQY
DKIA
>Anc90 MalDH_UL3
------------------------------------------------A-----------
----------------------------------------------I-------------
------------------------------------------------------------
---------------------E--------------------------------------
------------------------------D----I------------------------
---S
>Anc93 MalDH_CATGTR
MTKVSMVGAAGTVGAAAGYNIALRDIADELVFVDIPDKEDDTIGQAADANHGAAYDSNTT
IRQGGYEDTEGSDVVVITAGIPRQPGQTRIDLAGDNAPIMEDIGSSLAEYNDDFITVTTS
NPVDLLNRHLYETGDRAREKVIGFGGRLDSARFRYVISQRYDAPVQNVEATILGEHGDAQ
VPVFSKVRVNGQDLEFTDDEKDELLEELQTSAMNVIEKKGATEWGPATGVGHMVEAILRD
TGEVLPASVKLEGEFGHEDTAFGVPVKLGSNGVEEVVEWDLTEFERNQLGEAAEKLSEQY
DKIA
>Anc93 MalDH_UL3
------------------------------------------------------------
----------------------------------------------I-------------
------------------------------------------------------------
------------------------------------------------------------
-----------------------------------I------------------------
----
```

FIG.S4. Comparison Sequence alignment between CAT+GTR and UL3 ancestral sequences. In MalDH_UL3 lines, gaps ("-") indicate that the same amino acid is obtained using CAT+GTR and UL3 models, and letters indicate the amino acids differently inferred using UL3.

Species	[NaCl], M	DE/KR	DE/KR	pI
M. tindarius	0.5	38/25	1.52	4.7
M. barkeri	0.4	39/28	1.39	4.8
M. mazei	0.3	39/32	1.21	5.1
M. acetivorans	0.2	36/31	1.16	5.2
M. psychrophilus	ND	38/28	1.35	5.0
M. holandica	0.2	37/32	1.15	5.3
M. evestigatum	4.3	45/25	1.8	4.5
M. portucalensis	2.2	41/29	1.41	4.8
M. mahii	2.0	43/26	1.65	4.5
M. halophilus	1.5	44/23	1.91	4.5
M. zhilinae	0.6	43/28	1.53	4.7
M. methylutens	0.3	37/26	1.42	4.9

Table S4. Acidic over basic amino acid ratio calculated using Malate dehydrogenase sequence from Methanosarcinales. The number of amino acid is in the third column. The result is in the fourth column. The pI is also indicated. The split in two groups was done accordingly to Gonzalez-Ordenes *et al.*, 2018. Calculation done using https://web.expasy.org/cgi-bin/protparam/protparam

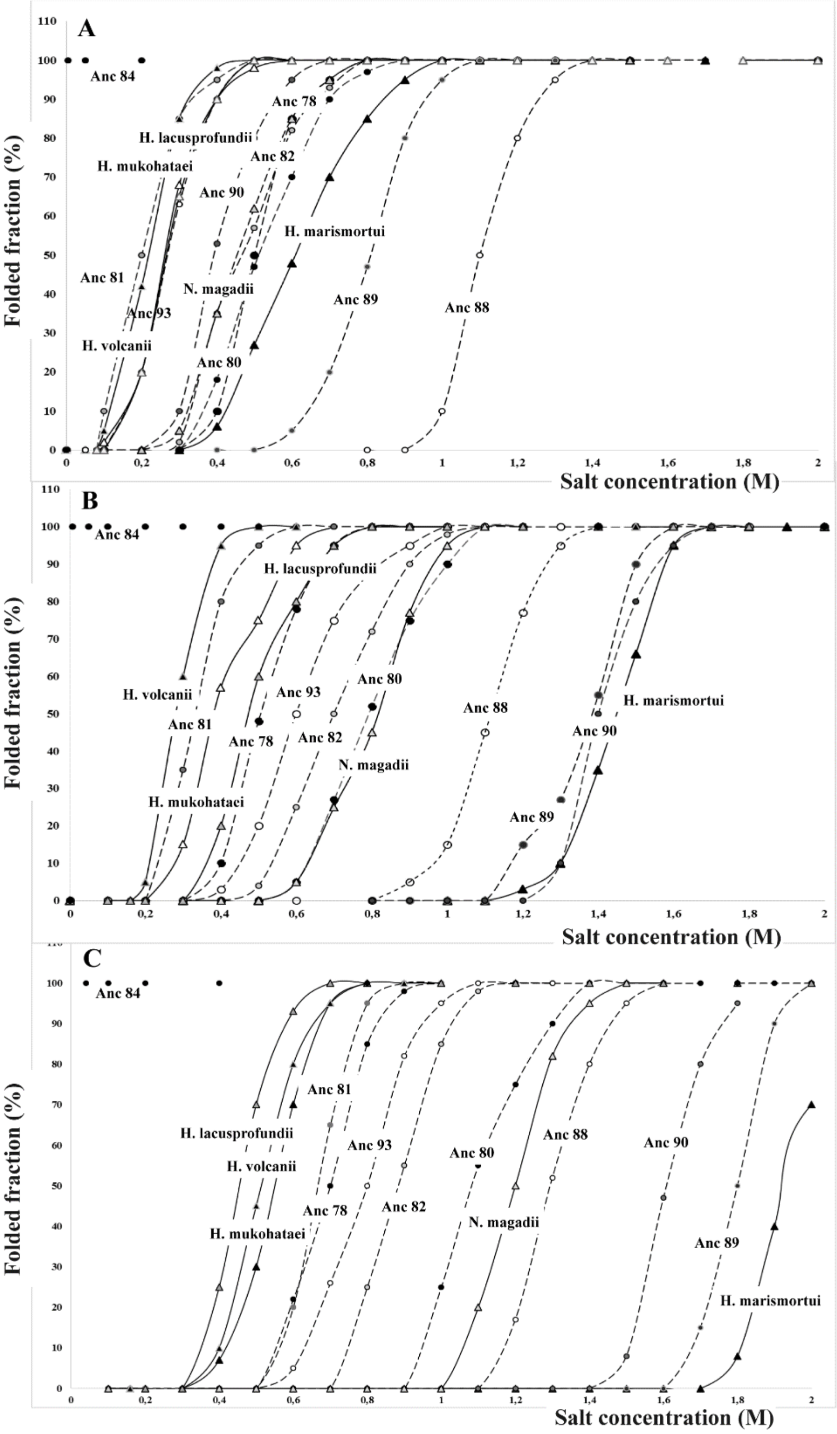

FIG.S5. Determination of the folded-unfolded transition of ancestral and modern MalDH. The measurement were done after 24 h of incubation at 25 °C in various concentration of different salts. (A) Potassium fluoride. (B) Potassium chloride. (C) Cesium chloride. Experimental details are given in the Materials and Methods. The data are normalized to the maximal values obtained for each enzyme prior incubation and stored in KCl 3.8 M. All the protein are stable at concentration higher than 2 M, in the three salt tested, this is why the (x) graph axis is truncated. As in our previous studies the folded fractions are given with a confident limit of +/- 5 % (Ebel *et al.* 1999; Irimia *et al.* 2003). For the sake of clarity, the errors bars are not shown. To avoid confusion with y-axis of the graph, the curve for Anc84 MalDH is not plotted.

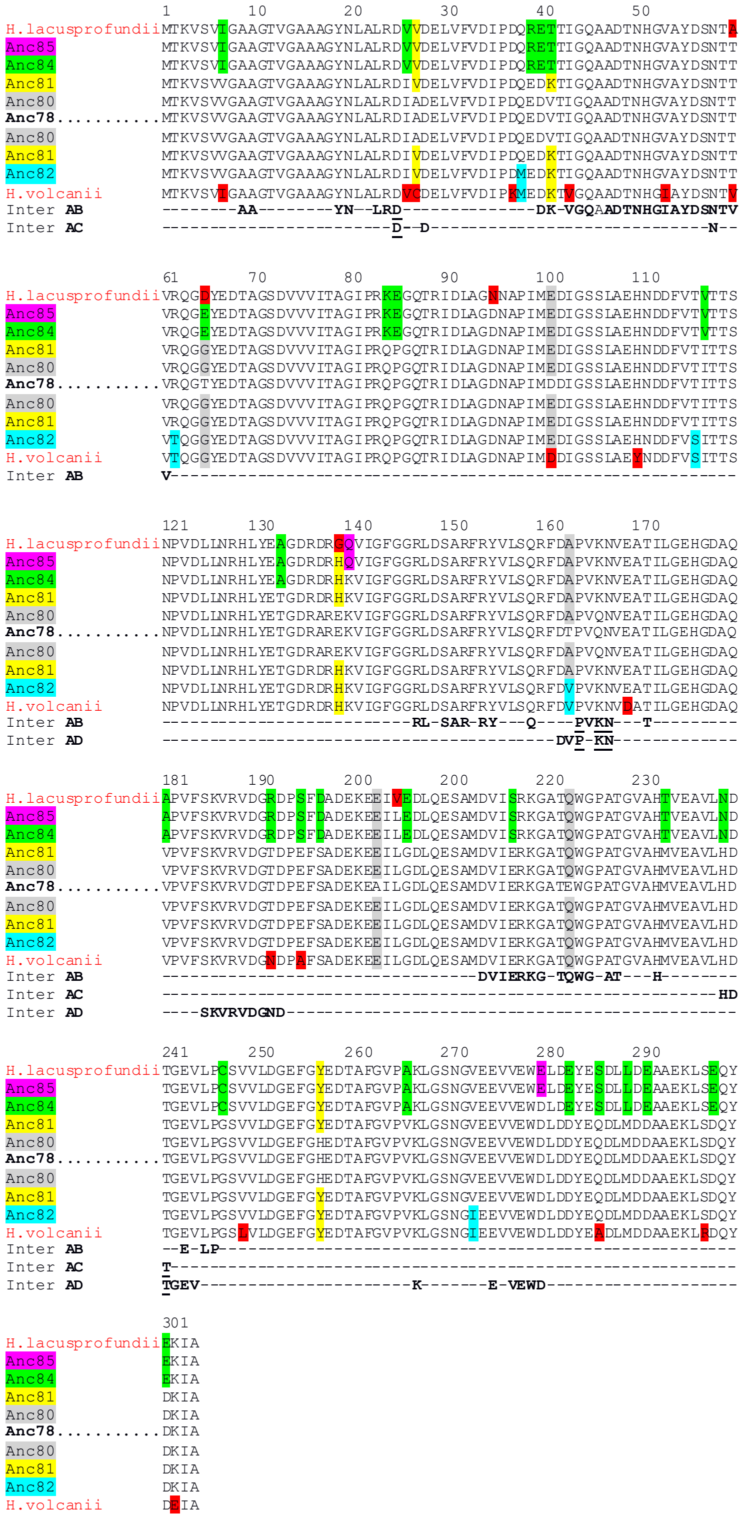

Fig.S6. Sequence alignment showing the amino acid replacement across modern MalDHs of *H lacusprofundii* and *H. volcanii* in group B, and their ancestors. Top of the alignment: ancestors Anc78 (root), Anc80 (group B and C LCA), Anc81 (group B LCA), Anc84 and Anc85 are successive MalDH sequences from the tree root MalDH to the *H. lacu* MalDH. Middle of the alignment: ancestors Anc78 (root), Anc80 (group B and C LCA), Anc81 (group B LCA), and Anc82 are successive MalDH sequences from the tree root MalDH to *H. volc* MalDH. Coloured amino acids in the MalDH sequences indicate the replacements compared to the root Anc78. Bottom of the alignment: residues

involved in the various interfaces AB, AD and AC are in bold. Calculation based on *Haloarcula marismortui* structure (2J5K) using PDBePISA. http://www.ebi.ac.uk/pdbe/pisa/. Underlined residues indicate that amino acids are involved in several interfaces.

```
                1        10        20        30        40        50
H.mukohataei    MTKVSVVGAAGTVGAAAGYNIALRDIADELVYVDIPDQEDVTIGQAADTNHGVAYDSNTT
Anc79           MTKVSVVGAAGTVGAAAGYNLALRDIADELVFVDIPDQEDVTIGQAADTNHGVAYDSNTT
Anc78...........MTKVSVVGAAGTVGAAAGYNLALRDIADELVFVDIPDQEDVTIGQAADTNHGVAYDSNTT
Anc80           MTKVSVVGAAGTVGAAAGYNLALRDIADELVFVDIPDQEDVTIGQAADTNHGVAYDSNTT
Anc88           MTKVSVVGAAGTVGAAAGYNIALRDIADELVFVDIPDQEDDTIGQAADTNHGVAYDSNTT
H.marismortui   MTKVSVVGAAGTVGAAAGYNIALRDIADEVVFVDIPDKEDDTVGQAADTNHGIAYDSNTR
Inter AB        --------AA--------YN--LRD--------------DK-VGQAADTNHGIAYDSNTV
Inter AC        ------------------------D--D-----------------------------N--

                61       70        80        90        100       110
H.mukohataei    IRQGTYEDTAGSDVVVITAGIPRQPGQTRIDLAGDNAPIMDDIGSSLAEHNDDFVTITTS
Anc79           VRQGTYEDTAGSDVVVITAGIPRQPGQTRIDLAGDNAPIMDDIGSSLAEHNDDFVTITTS
Anc78...........VRQGTYEDTAGSDVVVITAGIPRQPGQTRIDLAGDNAPIMDDIGSSLAEHNDDFVTITTS
Anc80           VRQGGYEDTAGSDVVVITAGIPRQPGQTRIDLAGDNAPIMEDIGSSLAEHNDDFVTITTS
Anc88           VRQGGYEDTAGSDVVVITAGIPRQPGQTRIDLAGDNAPIMEDIGSSLAEHNDDFVTITTS
H.marismortui   VRQGGYEDTAGSDVVVITAGIPRQPGQTRIDLAGDNAPIMEDIQSSLDEHNDDYISLTTS
Inter AB        V-----------------------------------------------------------

                121      130       140       150       160       170
H.mukohataei    NPVDLLNRHLYETGDRAREKVIGFGGRLDSARFRYVLAERFDTEVQNVEATILGEHGDAQ
Anc79           NPVDLLNRHLYETGDRAREKVIGFGGRLDSARFRYVLSERFDTEVQNVEATILGEHGDAQ
Anc78...........NPVDLLNRHLYETGDRAREKVIGFGGRLDSARFRYVLSQRFDTPVQNVEATILGEHGDAQ
Anc80           NPVDLLNRHLYETGDRAREKVIGFGGRLDSARFRYVLSQRFDAPVQNVEATILGEHGDAQ
Anc88           NPVDLLNRHLYETGDRAREKVIGFGGRLDSARFRYVLSQRFDAPVQNVEATILGEHGDAQ
H.marismortui   NPVDLLNRHLYEAGDRSREQVIGFGGRLDSARFRYVLSEEFDAPVQNVEGTILGEHGDAQ
Inter AB        --------------------------RL-SAR-RY---Q----PVKN---T---------
Inter AD        -----------------------------------------DVP-KN-------------

                181      190       200       200       220       230
H.mukohataei    VPVFSKVRIDGADPDFSADEKEAILGELQESAMDVIERKGATEWGPATGVAHMVEAVLRD
Anc79           VPVFSKVRVDGTDPEFSADEKEAILGDLQESAMDVIERKGATEWGPATGVAHMVEAVLHD
Anc78...........VPVFSKVRVDGTDPEFSADEKEAILGDLQESAMDVIERKGATEWGPATGVAHMVEAVLHD
Anc80           VPVFSKVRVDGTDPEFSADEKEEILGDLQESAMDVIERKGATQWGPATGVAHMVEAVLHD
Anc88           VPVFSKVRVDGTDPEFSADEKEELLGDLQESAMDVIERKGATQWGPATGVAHMVEAILHD
H.marismortui   VPVFSKVRVDGTDPEFSGDEKEQLLGDLQESAMDVIERKGATEWGPARGVAHMVEAILHD
Inter AB        ---------------------------------DVIERKG-TQWG-AT---H--------
Inter AC        ----------------------------------------------------------HD
Inter AD        ----SKVRVDGND-----------------------------------------------

                241      250       260       270       280       290
H.mukohataei    TGEVLPGSVVLDGEFGHEGTAFGVPVKLGSDGVEEVVEWDLDDYEQDLMAEASEKLAEQY
Anc79           TGEVLPGSVVLDGEFGHEDTAFGVPVKLGSDGVEEVVEWDLDDYEQDLMDDAAEKLSDQY
Anc78...........TGEVLPGSVVLDGEFGHEDTAFGVPVKLGSNGVEEVVEWDLDDYEQDLMDDAAEKLSDQY
Anc80           TGEVLPGSVVLDGEFGHEDTAFGVPVKLGSNGVEEVVEWDLDDYEQDLMDDAAEKLSDQY
Anc88           TGEVLPASVKLDGEFGHEDTAFGVPVKLGSNGVEEVVEWDLDDYEQDLMDDAAEKLSDQY
H.marismortui   TGEVLPASVKLEGEFGHEDTAFGVPVRLGSNGVEEIVEWDLDDYEQDLMADAAEKLSDQY
Inter AB        --E-LP------------------------------------------------------
Inter AC        T-----------------------------------------------------------
Inter AD        TGEV----------------------K-------E-VEWD--------------------

                301
H.mukohataei    DKIG
Anc79           DKIA
Anc78...........DKIA
Anc80           DKIA
Anc88           DKIA
H.marismortui   DKIS
```

Fig S7. Sequence alignment showing the amino acid replacement across modern MalDHs of *H. mukohataei* and *H. marismortui* in group A, and their ancestors. Top of the alignment: ancestors Anc78 (root) and Anc79 are successive MalDH sequences from the tree root MalDH to the *H. muko* MalDH. Middle of the alignment: ancestors Anc78 (root), Anc80 (group B and C LCA) and Anc88 are successive MalDH sequences from the tree root MalDH to *H. mari* MalDH. Colored amino acids in the MalDH sequences indicate the replacements compared to the root Anc78. Bottom of the alignment: residues involved in the various interfaces AB, AD and AC are in bold. Calculation based on *Haloarcula marismortui* structure (2J5K) using PDBePISA. http://www.ebi.ac.uk/pdbe/pisa/. Underlined residues indicate that amino acids are involved in several interfaces.

```
                    1        10        20        30        40        50
N.magadii           MTKVSVVGAAGTVGAAAGYNIALRDIADELVFVDIPDKEDDTIGQAADANHGAAYDSNTT
Anc94               MTKVSMVGAAGTVGAAAGYNIALRDIADELVFVDIPDKEDDTIGQAADANHGAAYDSNTT
Anc93               MTKVSMVGAAGTVGAAAGYNIALRDIADELVFVDIPDKEDDTIGQAADANHGAAYDSNTT
Anc90               MTKVSVVGAAGTVGAAAGYNIALRDIADELVFVDIPDKEDDTIGQAADTNHGAAYDSNTT
Anc89               MTKVSVVGAAGTVGAAAGYNIALRDIADELVFVDIPDQEDDTIGQAADTNHGVAYDSNTT
Anc88               MTKVSVVGAAGTVGAAAGYNIALRDIADELVFVDIPDQEDDTIGQAADTNHGVAYDSNTT
Anc80               MTKVSVVGAAGTVGAAAGYNLALRDIADELVFVDIPDQEDVTIGQAADTNHGVAYDSNTT
Anc78...............MTKVSVVGAAGTVGAAAGYNLALRDIADELVFVDIPDQEDVTIGQAADTNHGVAYDSNTT
Inter AB            --------AA--------YN--LRD--------------DK-VGQAADTNHGIAYDSNTV
Inter AC            ------------------------D--D-----------------------------N--

                    61       70        80        90        100       110
N.magadii           IRQGGYEATEGSDVVVITAGIPRQPGQTRIDLAGDNAPIMEDIGSSIAEYNDDFITVTTS
Anc94               IRQGGYEDTEGSDVVVITAGIPRQPGQTRIDLAGDNAPIMEDIGSSIAEYNDDFITVTTS
Anc93               IRQGGYEDTEGSDVVVITAGIPRQPGQTRIDLAGDNAPIMEDIGSSLAEYNDDFITVTTS
Anc90               IRQGGYEDTAGSDVVVITAGIPRQPGQTRIDLAGDNAPIMEDIGSSLAEHNDDFVTVTTS
Anc89               VRQGGYEDTAGSDVVVITAGIPRQPGQTRIDLAGDNAPIMEDIGSSLAEHNDDFVTVTTS
Anc88               VRQGGYEDTAGSDVVVITAGIPRQPGQTRIDLAGDNAPIMEDIGSSLAEHNDDFVTITTS
Anc80               VRQGGYEDTAGSDVVVITAGIPRQPGQTRIDLAGDNAPIMEDIGSSLAEHNDDFVTITTS
Anc78...............VRQGTYEDTAGSDVVVITAGIPRQPGQTRIDLAGDNAPIMDDIGSSLAEHNDDFVTITTS
Inter AB            V-----------------------------------------------------------

                    121      130       140       150       160       170
N.magadii           NPVDLLNRHLYEAGNRARDKVIGFGGRLDSARFRYVISQRYDVPVQNVEATILGEHGDAQ
Anc94               NPVDLLNRHLYETGDRAREKVIGFGGRLDSARFRYVISQRYDAPVQNVEATILGEHGDAQ
Anc93               NPVDLLNRHLYETGDRAREKVIGFGGRLDSARFRYVISQRYDAPVQNVEATILGEHGDAQ
Anc90               NPVDLLNRHLYETGDRAREKVIGFGGRLDSARFRYVISQRFDAPVQNVEATILGEHGDAQ
Anc89               NPVDLLNRHLYETGDRAREKVIGFGGRLDSARFRYVISQRFDAPVQNVEATILGEHGDAQ
Anc88               NPVDLLNRHLYETGDRAREKVIGFGGRLDSARFRYVLSQRFDAPVQNVEATILGEHGDAQ
Anc80               NPVDLLNRHLYETGDRAREKVIGFGGRLDSARFRYVLSQRFDAPVQNVEATILGEHGDAQ
Anc78...............NPVDLLNRHLYETGDRAREKVIGFGGRLDSARFRYVLSQRFDTPVQNVEATILGEHGDAQ
Inter AB            --------------------------RL-SAR-RY---Q----PVKN---T---------
Inter AD            -----------------------------------------DVP-KN-------------

                    181      190       200       200       220       230
N.magadii           VPVFSKVRVNGQDLEFTDDEKDELLEELQTSAMNVIEKKGATEWGPATGVGHMVEAILRD
Anc94               VPVFSKVRVNGQDLEFTDDEKDELLEELQTSAMNVIEKKGATEWGPATGVGHMVEAILRD
Anc93               VPVFSKVRVNGQDLEFTDDEKDELLEELQTSAMNVIEKKGATEWGPATGVGHMVEAILRD
Anc90               VPVFSKVRVDGQDPEFDEDEKDELLEELQTSAMNVIEKKGATQWGPATGVGHMVEAILRD
Anc89               VPVFSKVRVDGQDPEFDEDEKEELLGELQESAMNVIEKKGATQWGPATGVAHMVEAILRD
Anc88               VPVFSKVRVDGTDPEFSADEKEELLGDLQESAMDVIERKGATQWGPATGVAHMVEAILHD
Anc80               VPVFSKVRVDGTDPEFSADEKEEILGDLQESAMDVIERKGATQWGPATGVAHMVEAVLHD
Anc78...............VPVFSKVRVDGTDPEFSADEKEAILGDLQESAMDVIERKGATEWGPATGVAHMVEAVLHD
Inter AB            ---------------------------------DVIERKG-TQWG-AT---H--------
Inter AC            ----------------------------------------------------------HD
Inter AD            ----SKVRVDGND-----------------------------------------------

                    241      250       260       270       280       290
N.magadii           TGEVLPASVTLEGEFGHEDTAFGVPVKLGSNGVEEVVEWDLTEYERNQLGEAAEKLSDQY
Anc94               TGEVLPASVKLEGEFGHEDTAFGVPVKLGSNGVEEVVEWDLTEFERNQLGEAAEKLSEQY
Anc93               TGEVLPASVKLEGEFGHEDTAFGVPVKLGSNGVEEVVEWDLTEFERNQLGEAAEKLSEQY
Anc90               TGEVLPASVKLEGEFGHEDTAFGVPVKLGSNGVEEVVEWDLTEYERNQLGEAAEKLSEQY
Anc89               TGEVLPASVKLDGEFGHEDTAFGVPVKLGSNGVEEVVEWDLTDYEQDQLDEAAEKLSEQY
Anc88               TGEVLPASVKLDGEFGHEDTAFGVPVKLGSNGVEEVVEWDLDDYEQDLMDDAAEKLSDQY
Anc80               TGEVLPGSVVLDGEFGHEDTAFGVPVKLGSNGVEEVVEWDLDDYEQDLMDDAAEKLSDQY
Anc78...............TGEVLPGSVVLDGEFGHEDTAFGVPVKLGSNGVEEVVEWDLDDYEQDLMDDAAEKLSDQY
Inter AB            --E-LP------------------------------------------------------
Inter AC            T-----------------------------------------------------------
Inter AD            TGEV----------------------K-------E-VEWD--------------------

                    301
N.magadii           DKIA
Anc94               DKIA
Anc93               DKIA
Anc90               DKIA
Anc89               DKIA
Anc88               DKIA
Anc80               DKIA
Anc78...............DKIA
```

Fig. S8. Sequence alignment showing the amino acid replacement across modern MalDH of *H magadii* in group C, and its ancestors. Top of the alignment: ancestors Anc78 (root), Anc80 (group B and C LCA), Anc88 (group B + *H. marismortui* LCA), Anc89 (group C LCA), Anc90, Anc93 and Anc94 are successive MalDH sequences from the tree root MalDH to the *H. maga* MalDH. Coloured amino acids in the MalDH sequences indicate the replacements compared to the root Anc78. Bottom of the alignment: residues involved in the various interfaces AB, AD and AC are in bold. Calculation based on *Haloarcula marismortui* structure (2J5K) using PDBePISA. http://www.ebi.ac.uk/pdbe/pisa/. Underlined residues indicate that amino acids are involved in several interfaces.

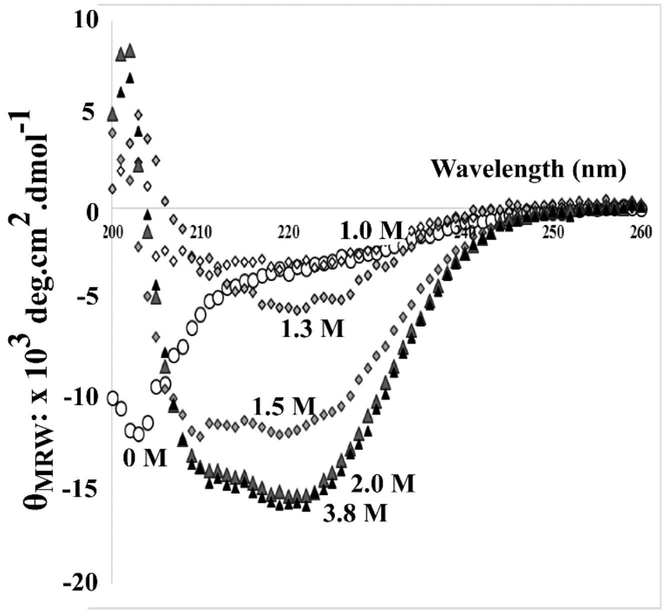

FIG. S9. Circular spectroscopy spectra of *H. mari* MalDH recorded after 24H of incubation at 25°C in various concentration of KCl supplemented with Tris-HCl 50 mM pH 7.0. Spectra in 3.8 and 2 M KCl, are typical of well folded protein. Below 2M, the protein deactivates and unfolds as shown by the strong change in molar ellipticity values.

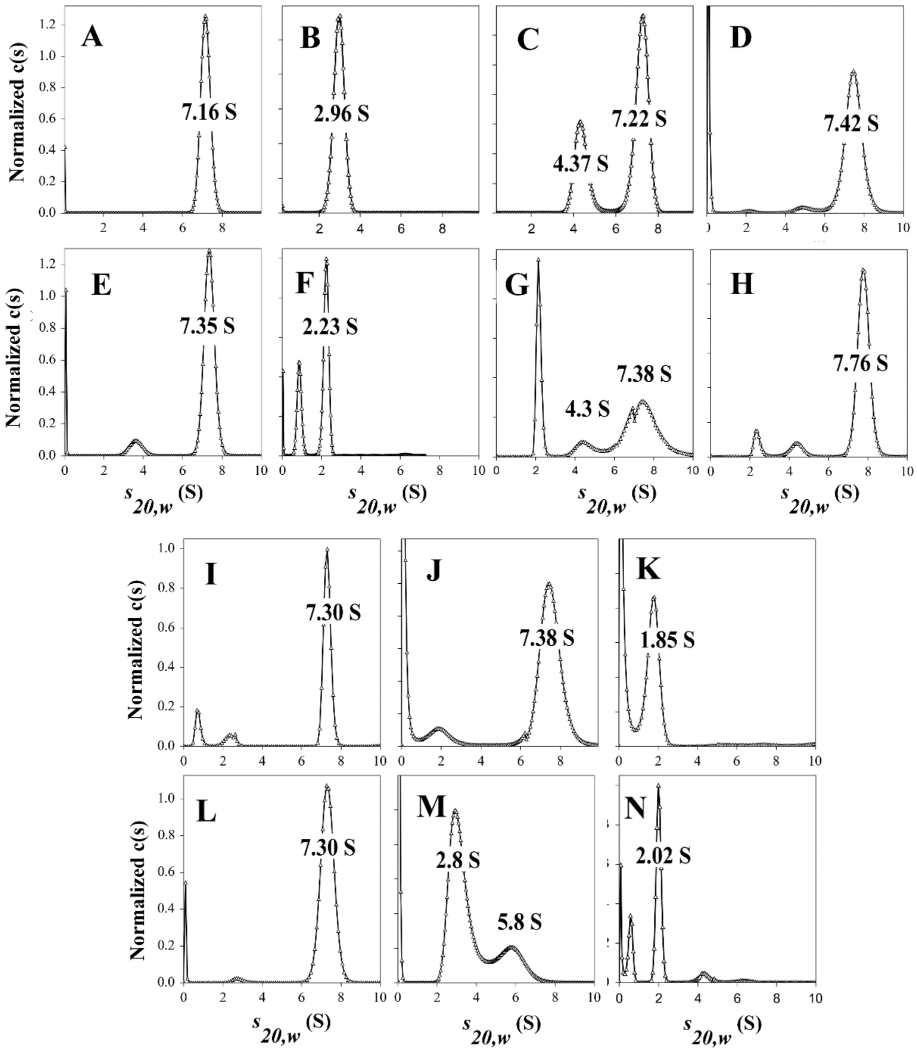

FIG.S10. Sedimentation coefficient of modern and ancestral MalDH recorded after 24 h of incubation in various salt conditions. All the solutions were buffered with 50 mM Tris-HCl pH7. The various panels show the sedimentation coefficient distribution calculated the sofwares Sedfit (https://sedfitsedphat.nibib.nih.gov/software) and Gussi (http://biophysics.swmed.edu/MBR/software). (A) Data on *H. volc* MalDH in 1.0 M KCl; (B) 0.2 M KCl; 0.2 M KF and (D) 0.1 M KCl + 0.050 M $MgCl_2$. (E) Data on Anc80 MalDH in 2.0 M KCl; (F) 0.6 M KCl; (G) 0.6 M KF and (H) 0.1 M KCl + 0.050 M $MgCl_2$. (I) Data on Anc89 MalDH 2.0 M KCl; (J) 0.6 M KCl + 0.5 M $MgCl_2$ and (K) 0.6 M KCl + 0.05 M $MgCl_2$. (L) Data on Anc90 MalDH in 2.0 M KCl and (M) 0.6 M KCl + 0.5 M $MgCl_2$. The $s_{20,W}$ values of 5.8 S is very likely due to a mixture of various dimers and tetramer in rapid equilibrium. (N) Data on *N. maga* MalDH in 0.6 M KCl + 0.05 M $MgCl_2$.

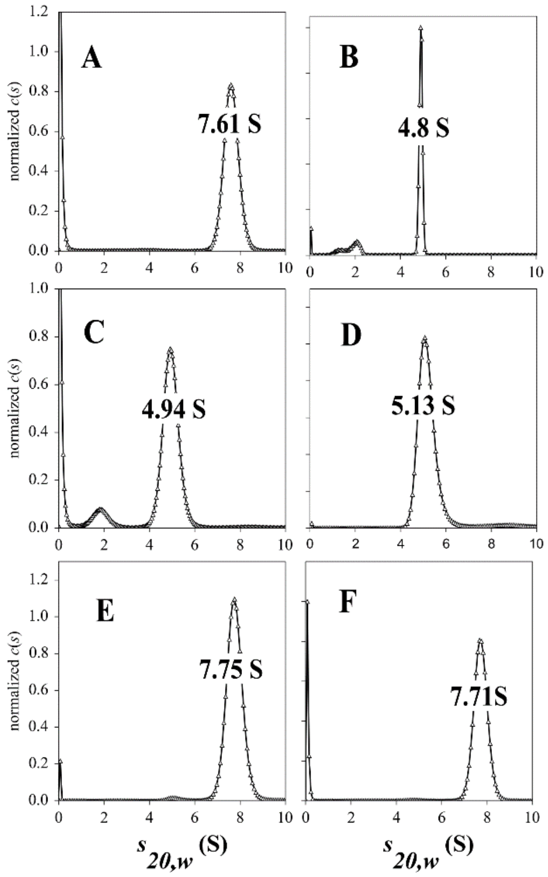

FIG. S11. Oligomeric variation of Anc84 MalDH in various salt conditions. In all conditions the sample were buffered with 50 mM Tris-HCl pH7. The panels show the sedimentation coefficient distribution of Anc84 MalDH (in Svedberg) calculated using the sofwares Sedfit (https://sedfitsedphat.nibib.nih.gov) and Gussi (http://biophysics.swmed.edu/MBR). (A) 1.0 M KCl; (B) 0.06 M KCl; (C) 0.1 M KCl; (D) 0.1 M KF; (E) 0.1 M KCl + 0.05 M $MgCl_2$ and (F) sample dialyzed in 0.05 M $MgCl_2$.

References.

Aouad M, Taib N, Oudart A, lecocq M, Gouy M, Brochier-Armanet C. 2018. Extreme halophilic archaea derive from two disctinct methanogen Class II lineages. *Mol Phylo Evol.* 127:46-54.

Becker EA, Seitzer PM, Tritt A, Larsen D, Krusor M, Yao AI, Wu D, Madern D, Eisen JA, Darling AE, Facciotti MT. 2014. Phylogenetically driven sequencing of extremely halophilic archaea reveals strategies for static and dynamic osmo-response. *PLos Genet.*10(11):e1004784.

Brinkmann H, van der Giezen M, Zhou Y, de Raucourt GP, Philippe H. 2005. An empirical assessment of Long Branch Attraction Artefacts in Deep Eukaryotic. *Phylogenomics Syst Biol*. 54(5):743-757.

Brochier-Armanet C, Forterre P, Gribaldo S. 2011. Phylogeny and evolution of the Archaea: one hundred genomes later. *Curr Op Microb*. 14(3):274-281.

Ebel C, Faou P, Kernel B, Zaccai G. 1999. Relative role of anions and cations in the stabilization

of halophilic malate dehydrogenase. *Biochemistry*. 38(28):9039-9047.
Groussin M, Boussau B, Gouy M. 2013. A Branch-Heterogeneous Model of Protein Evolution for Efficient Inference of Ancestral Sequences. *Syst Biol.* 62(4):523–538.
Guindon S, Dufayard JF, Lefort V, Anisimova M, Hordijk W, Gascuel O. 2010. New algorithms and methods to estimate maximum-likelihood phylogenies: assessing the performance of PhyML 3.0. *Syst Biol.* 59(3):307-321.
Irimia A, Ebel C, Madern D, Richard SB, Cosenza LW, Zaccai G, Vellieux FMD. 2003. The oligomeric states of *Haloarcula marismortui* malate dehydrogenase are modulated by solvent components as shown by crystallographic and biochemical studies. *J Mol Biol.* 326(3):859-873.
Lartillot N, Lepage T, Blanquart S. 2009. PhyloBayes 3. A Bayesian software package for phylogenetic reconstruction and molecular dating. *Bioinformatics*. 25(17):2286-2288.
Lartillot N, Philippe H. 2004. A Bayesian mixture model for across-site heterogeneities in the amino-acid replacement process. *Mol Biol Evol.* 21(6):1095-2004.
Le SQ, Gascuel O. 2008. An Improved General Amino Acid Replacement Matrix. *Mol Biol Evol.* 25(7):1307-1320.
Löytynoja A, Goldman N. 2008. Phylogeny-aware gap placement prevents errors in sequence alignment and evolutionary analysis. *Science*. 320(5883):1632-1635.
Miele V, Penel S, Duret L. 2011. Ultra-fast sequence clustering from similarity networks with SiLiX. *BMC Bioinformatics* 12:116.
Nguyen TH, Ranwez V, Pointet S, Chifolleau AMA, Doyon JP, Berry V. 2013. Reconciliation and local gene tree rearrangement can be of mutual profit. *Algorithms for Molecular Biology* 8(1):12.
Philippe H, Delsuc F, Brinkmann H, Lartillot N. 2005. Phylogenomics. *Annu Rev Ecol Evol Syst*. 36:541-562.
Szöllosi GJ, Rosikiewicz W, Boussau B, Tannier E, Daubin V. 2013a. Efficient exploration of the space of reconciled gene trees. *Syst Biol.* 62(6):901-912.
Szöllosi GJ, Tannier E, Lartillot N, Daubin V. 2013b. Lateral Gene Transfer from the Dead. *Syst Biol.* 62(3):386–397.
